# STAT3-coordinated migration facilitates the dissemination of diffuse large B-cell lymphomas

**DOI:** 10.1038/s41467-018-06134-z

**Published:** 2018-09-12

**Authors:** Yi-Ru Pan, Chih-Cheng Chen, Yu-Tien Chan, Hsiao-Jung Wang, Fan-Tso Chien, Yeng-Long Chen, Jing-Lan Liu, Muh-Hwa Yang

**Affiliations:** 1 Institute of Clinical Medicine, National Yang-Ming University, Taipei, 11221 Taiwan; 2 Division of Hematology and Oncology, Chang Gung Memorial Hospital, Chiayi, 61363 Taiwan; 3 College of Medicine, Chang Gung University, Tao-Yuan, 33302 Taiwan; 4 Division of Experimental Surgery, Department of Surgery, Taipei Veterans General Hospital, Taipei, 11217 Taiwan; 5 Institute of Physics, Academia Sinica, Taipei, 11529 Taiwan; 6 Department of Pathology, Chang Gung Memorial Hospital, Chiayi, 61363 Taiwan; 7 Cancer Progression Research Center, National Yang-Ming University, Taipei, 11221 Taiwan; 8 Division of Medical Oncology, Department of Oncology, Taipei Veterans General Hospital, Taipei, 11217 Taiwan

## Abstract

The motile characteristics and mechanisms that drive the dissemination of diffuse large B-cell lymphoma (DLBCL) are elusive. Here, we show that DLBCL initiates dissemination through activating STAT3-mediated amoeboid migration. Mechanistically, STAT3 activates *RHOH* transcription, which competes with the RhoGDP dissociation inhibitor RhoGDIγ to activate RhoA. In addition, activated STAT3 regulates microtubule dynamics and releases ARHGEF2 to activate RhoA. Both the JAK inhibitor ruxolitinib and the microtubule stabilizer Taxol suppress DLBCL cell dissemination in vivo. A clinical DLBCL sample analysis shows that STAT3-driven amoeboid movement is particularly important for the transition from stage I to stage II. This study elucidates the mechanism of DLBCL dissemination and progression and highlights the potential of combating advanced DLBCL with a JAK/STAT inhibitor or microtubule stabilizer to reduce DLBCL motility; these findings may have a great impact on the development of patient-tailored treatments for DLBCL.

## Abstract (web summary)

The mechanism underlying the dissemination of diffuse large B-cell lymphoma (DLBCL) is unclear. Here, the authors show that STAT3 controls amoeboid migration in DLBCL via the transcriptional activation of *RHOH*, which then releases RhoA from RhoGDIγ-mediated suppression, or via regulating microtubule dynamics to activate RhoA.

## Introduction

Diffuse large B-cell lymphoma (DLBCL), an aggressive lymphoid malignancy that arises primarily from mature B lymphocytes in the germinal center of the lymph node, is the most prevalent type of lymphoma and accounts for 30% of all non-Hodgkin’s lymphomas in adults^1^. The clinical presentation of DLBCL is a single, rapidly enlarged mass (localized disease) or multiple lymphadenopathies (disseminated disease)^1^. During dissemination, DLBCL cells lack focal contacts and have a high level of plasticity^2^. DLBCL treatment yields an excellent response to the localized disease. Nevertheless, the response is reduced significantly in the disseminated disease^3^, indicating the necessity of targeting disseminated lymphoma cells in advanced-stage cases. However, most current therapies overlook the impact of DLBCL cell dissemination and focus mainly on inhibiting proliferation and inducing apoptosis in lymphoma cells.

The deregulation of normal B cell signals that sustain growth and survival is commonly noted in DLBCL. Myc, B-cell lymphoma 6 (BCL6), and B-cell lymphoma 2 (BCL-2) are commonly overexpressed following chromosomal translocation, resulting in the abnormal proliferation of lymphoma cells^4–6^. Constitutive activation of the NF-κB pathway is observed predominantly in activated B-cell (ABC)-type DLBCL^7^. Recent studies have highlighted the importance of deregulated cytokine-mediated signaling pathways in DLBCL progression. Activation of the transcription factor signal transducer and activator of transcription 3 (STAT3) correlates with a worse DLBCL prognosis^8^. Increased levels of interleukin 6 (IL-6) and interleukin 10 (IL-10), the major upstream cytokines of STAT3^9^, are associated with a poor DLBCL prognosis^10^. Although the oncogenic signals that sustain DLBCL cell proliferation and survival have been studied extensively, the link between the proliferation/survival signals and mechanisms of DLBCL cell dissemination remains elusive.

Amoeboid movement, which refers to the movement of the *Dictyostelium discoideum* amoeba, is a type of protease-independent movement that is characterized by low adhesion force and high actomyosin contractility^11^. Compared to cells with mesenchymal movement, another type of single cell movement, amoeboid-type cells move faster in three-dimensional (3D) culture systems^12^. The RhoA-Rho-associated protein kinase (ROCK)-myosin axis is the most well-known mechanism of cell contractility and is the major signaling pathway that induces amoeboid movement^13,14^. Amoeboid movement has been described as the major movement method for T-lymphocytes and normal hematopoietic cells^15^. In addition, amoeboid movement has been observed in different types of cancer cells^16^. However, the clinical impact and driving mechanism of amoeboid movement in DLBCL are unclear.

In this study, we describe the impact of amoeboid movement on DLBCL dissemination and the underlying mechanism. We show that STAT3 coordinates DLBCL movement through activating STAT3, which in turn activates *RHOH* or regulates microtubule dynamics to activate RhoA. Inhibiting JAK/STAT3 activity or intercepting microtubule assembly suppresses DLBCL migration. These findings provide valuable information regarding the development of advanced-stage DLBCL.

## Results

### Amoeboid movement is critical for DLBCL early dissemination

**Fig. 1:**
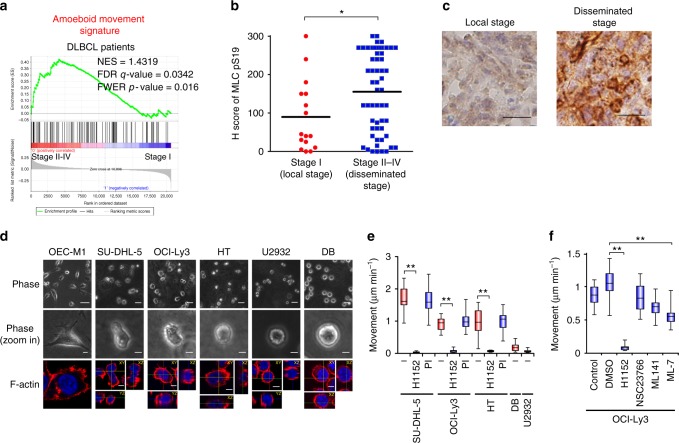
Amoeboid movement is involved in the early dissemination of DLBCL. **a** GSEA for examining the association between the amoeboid movement signature and gene expression array from DLBCL patients with stage II–IV vs. stage I (GSE11318). **b** Distribution of the H score for MLC S19 phosphorylation (MLCpS19) from DLBCL patients. *n* = 72. **c** Representative results of immunohistochemical staining for MLC S19 phosphorylation. Scale bar = 20 μm. **d** SU-DHL-5, OCI-Ly3, HT, U2932, and DB cells were grown in collagen gels and OEC-M1 cells were grown on top of collagen gels overnight. Upper and middle: representative phase-contrast images. Scale bar = 50 μm (Upper) or 10 μm (middle). Lower: immunofluorescent staining for F-actin (red). Blue, nuclei. Scale bar = 5 μm. **e** Quantification of movement speeds of lymphoma cell lines treated with inhibitors (*n* = 20). PI: proteinase inhibitor. **f** Quantification of movement speeds of OCI-Ly3 cells treated with inhibitors (*n* = 20). *P < 0.05, **P < 0.005 by Student’s *t*-test; Kolmogorov–Smirnov tests for GSEA. See also Supplementary Fig. 1 and Supplementary Movie 1. See Supplementary Table 1 for patient demographics in **b** and **c**; Supplementary Data 2 for amoeboid signature in **a**; Supplementary Table 4 for working concentration of inhibitors in **e** and **f**

In this study, we investigated the mechanism of DLBCL cell dissemination. We first confirmed the involvement of amoeboid movement in the dissemination of DLBCL. Gene set enrichment analysis (GSEA) showed that the gene expression signature of amoeboid movement, but not mesenchymal movement, was associated with DLBCL Ann Arbor stage II–IV, but not stage I (Fig. 1a and Supplementary Fig. 1a). A significant increase in the phosphorylated myosin light chain (MLC) levels, which indicates the activation of Rho-ROCK signaling and is a marker for amoeboid movement^17^, was observed in stage II–IV DLBCL patient samples (Fig. 1b, c, Supplementary Fig. 1b and Supplementary Table 1, 2), which supports the involvement of amoeboid movement in the early dissemination of DLBCL. Next, we investigated amoeboid movement in DLBCL using trajectory tracking and in vivo monitoring. Compared to the squamous cell carcinoma (SCC) cell line OEC-M1, which moves using the mesenchymal mechanism^18^, the DLBCL cell lines SUDHL-5, OCI-Ly3, HT, U2932, and DB displayed an amoeboid morphology in 3D collagen gels (Fig. 1d, Supplementary Fig. 1c and Supplementary Movie 1). Suppressing ROCK activity (by H1152) but not protease activity diminished the migration of DLBCL cells (Fig. 1e). Inhibiting ROCK activity (by H1152), suppressing MLC phosphorylation (by ML-7), or repressing myosin II activity (by blebbistatin) reduced amoeboid movement (Fig. 1f and Supplementary Fig. 1d, e).

**Fig. 2:**
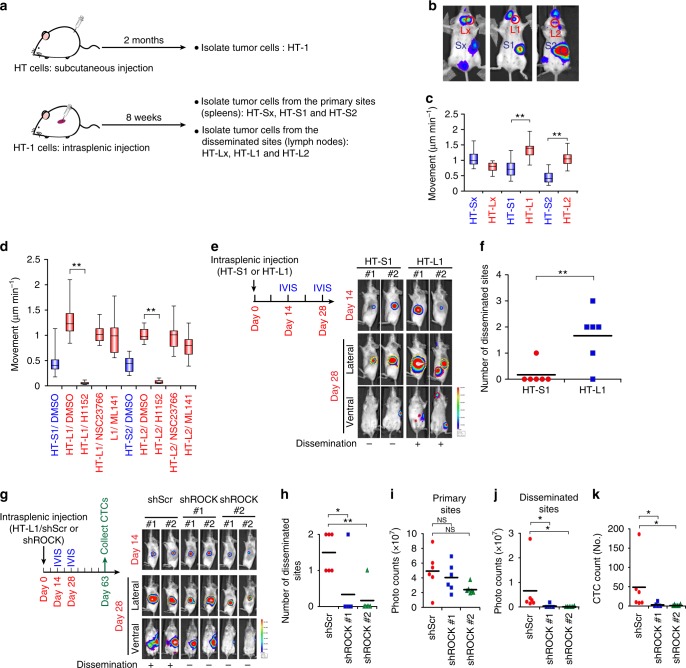
Amoeboid movement mediates DLBCL dissemination in vivo. **a** A schema for showing the establishment of DLBCL sublines with increased tumorigenic and disseminating capability. **b** Mice experiments for generating the HT sublines. HT-1 subline cells were injected into the splenic parenchyma, and the bioluminescent signals were detected on day 56. **c** Quantification of movement speeds of different sublines (*n* = 20). **d** Quantification of movement speeds of different sublines treated with corresponding inhibitors (*n* = 20). **e** Investigation of the in vivo dissemination of the HT-S1 or HT-L1 sublines. Cells were injected into the splenic parenchyma. Mice bioluminescent signals on indicated days were detected (*n* = 6; other 4 mice in Supplementary Fig. 2d). **f** Quantification of disseminated numbers (red dotted lines) in **e** and Supplementary Fig. 2d. **g** Investigation of the in vivo dissemination of HT-L1 receiving shRNAs specific to ROCK I (shROCK; clones #1, #2) or a scramble sequence (shScr). The cells were injected into the splenic parenchyma. Mice bioluminescent signals on indicated days were detected (*n* = 6; other 4 mice in Supplementary Fig. 2h). **h** Quantification of the numbers disseminated lesions in **g** and Supplementary Fig. 2h. **i**, **j** Quantification of bioluminescent intensity of **g** and Supplementary Fig. 2h from the primary sites (yellow dotted line circled area in photos)(**i**) and the disseminated sites (red dotted lines circled area in photos) (**j**). NS, not significant. **k** Quantification of the CTCs (circulating tumor cells) collected from the mice described in panel g (*n* = 6). *P < 0.05, **P < 0.005 by Student’s *t-*test. See also Supplementary Fig. 2 and Supplementary Movie 2. See Supplementary Table 4 for working concentration of inhibitors

We further investigated whether amoeboid movement contributed to DLBCL cell dissemination in vivo. We first inoculated the DLBCL cell line HT into subcutaneous regions of SCID/beige mice to enhance in vivo tumorigenicity. The tumor cells were harvested and cultivated as a subline named HT-1 (Fig. 2a). Next, we inoculated the HT-1 cells into the spleens of mice. Lymphoma cells disseminated after 8 weeks (Fig. 2b). We harvested tumor cells from the primary and metastatic sites and cultivated them to establish sublines. Three pairs of sublines were thus generated (HT-Sx/HT-Lx, HT-S1/HT-L1, and HT-S2/HT-L2). We then examined the motility of these sublines in a 3D collagen gel. Two of the three sublines established from the disseminated sites exhibited higher motility (HT-L1 and HT-L2) than the sublines from the primary sites (HT-S1 and HT-S2) (Fig. 2b, c and Supplementary Movie 2). Two pairs of the sublines (HT-L1/HT-S1 and HT-L2/HT-S2) were subjected to cDNA microarray and gene ontology analyses to assess the signals that regulate DLBCL cell motility. Of the activated signals in the migratory sublines, most signals were related to cell survival and metabolism. Small GTPase-mediated signals were found to be the candidate signals regulating DLBCL motility (Supplementary Fig. 2a and Supplementary Data 1). Moreover, of the major small GTPases, the activity of RhoA, which drives amoeboid movement, was increased in the sublines with higher motility^13^ (Supplementary Fig. 2b). Inhibiting ROCK activity, a downstream effector of RhoA, suppressed the motility of the HT-L1/HT-L2 sublines (Fig. 2d). Next, we investigated whether amoeboid movement contributed to DLBCL cell dissemination in vivo. The HT-L1 and HT-S1 sublines were injected into the spleens of mice and incubated for 4 weeks. Though HT-L1 did not have a higher proliferation rate compared with HT-S1 in vitro (Supplementary Fig. 2c), HT-L1 had a high capacity for dissemination in the mice (Fig. 2e, f and Supplementary Fig. 2d). ROCK knockdown in HT-L1 cells attenuated tumor cell dissemination and the number of circulating tumor cells (CTCs) without significantly affecting cellular proliferation and primary tumor growth (Fig. 2g–k and Supplementary Fig. 2e-h). These results suggest that Rho-ROCK axis-mediated amoeboid movement is critical for the early dissemination of DLBCL.

### JAK-STAT3 signaling is the major pathway in DLBCL movement

**Fig. 3:**
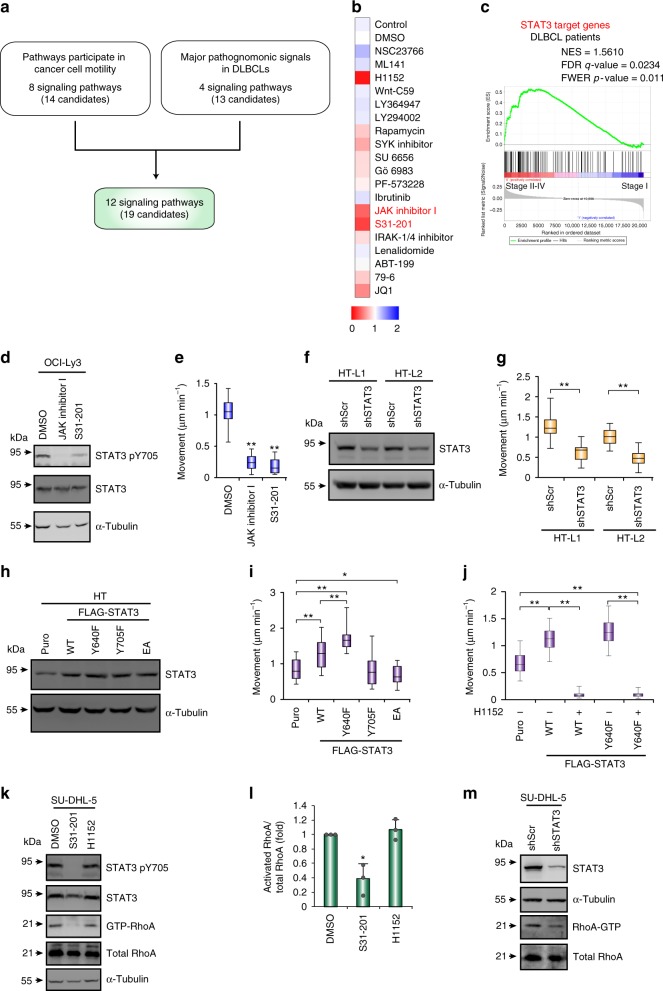
JAK-STAT3 signaling is the major pathway mediating cell movement in DLBCL. **a** A schema for showing the mining process for identification of the signaling pathways that regulate amoeboid movement in DLBCL. See Supplementary Table 3 for the information and references for the signal pathways. **b** A heat map showing the relative cell movement of SU-DHL-5 cells upon the treatment of inhibitors. The velocity of the cells was measured and expressed as fold relative the level control cells. Red, decreased motility level; blue, increased motility level. **c** A GSEA plot for showing the association between STAT3-regulated signature (Supplementary Data 2) and DLBCL stage I vs. II–IV (GSE11318). **d** Western blots for showing the efficacy of OCI-Ly3 cells treated with indicated inhibitors. **e** Quantification of movement speeds of inhibitor treated OCI-Ly3 cells (*n* = 20). **f** Western blots for showing the knockdown efficacy of HT-L1 and HT-L2 sublines receiving shRNAs specific to STAT3 (shSTAT3) or scramble (shScr). **g** Quantification of movement speeds of the cells described in panel f (*n* = 30). **h** Western blots for showing the transfection efficacy of HT cells expressing FLAG-STAT3 WT and its mutants. **i** Quantification of movement speeds of cells described in panel h (*n* = 20). **j** Quantification of movement speeds of HT cells expressing STAT3 WT, Y640F or control vector (puro) treated H1152 or DMSO (*n* = 30). **k**–**l** Representative results of western blots and pull down assay for showing the level of Y705-phosphorylated STAT3, STAT3, α-tubulin, active RhoA, and total RhoA in SU-DHL-5 cells under the treatment of different inhibitors for 6 h (**k**). Quantification of RhoA activity from three independent experiments (**l**). Data represent mean ± S.D. **m** Western blots and pull down assay for showing the level of STAT3, α-tubulin, active RhoA, and total RhoA in SU-DHL-5 cells receiving shRNA against STAT3 or a scramble sequence. *P < 0.05, **P < 0.005 by Student’s *t*-test; Kolmogorov–Smirnov tests for GSEA. See also Supplementary Fig. 3 and Supplementary Movie 3. See Supplementary Table 4 for working concentration of inhibitors

Next, we determined the major signaling pathway(s) controlling amoeboid movement in DLBCL. We treated DLBCL cells with a panel of inhibitors that suppressed the known oncogenic pathways in DLBCL or cell motility in cancer cells (Fig. 3a and Supplementary Table 3). Before measuring cell movement, we confirmed that these inhibitors did not induce cell apoptosis during the assay (Supplementary Fig. 3a). In addition to the ROCK inhibitor H1152, the pan-JAK (Janus kinase) inhibitor JAK inhibitor I, the STAT3 inhibitor S31-201, and the bromodomain protein inhibitor JQ1 also reduced amoeboid movement (Fig. 3b, Supplementary Fig. 3b and Supplementary Movie 3). Here, we focused on the JAK-STAT3 pathway because STAT3 is a major prognostic indicator of DLBCL^8^, and STAT3 has been shown to be involved in amoeboid movement in melanoma^19^. A significant association between the STAT3 signature and disseminated DLBCL (≥ stage II) was revealed by GSEA (Fig. 3c). Both the pan-JAK inhibitor and STAT3 inhibitor suppressed amoeboid movement (Fig. 3d, e, Supplementary Fig. 3c, d and Supplementary Movie 3). The phosphorylation of STAT3 Y705 was higher in the high-migratory HT-L1 and HT-L2 sublines than in the primary HT-S1 and HT-S2 sublines (Supplementary Fig. 3e). Knocking down STAT3 in HT-L1 and HT-L2, as well as SU-DHL-5 cells significantly attenuated amoeboid movement (Fig. 3f, g and Supplementary Fig. 3f, g, i, j) and reduced the probability of one-step displacement (Supplementary Fig. 3h). The ectopic expression of wild-type (WT) STAT3 or the gain-of-function STAT3 mutant Y640F^20^ increased amoeboid movement in HT cells. In contrast, overexpression of the JAK phosphorylated site Y705F STAT3 mutant or E434/435A mutant (EA), which are defective in DNA binding^21^, did not increase amoeboid movement (Fig. 3i). Inhibiting cyclin D1, a major target of STAT3 that mediates cellular proliferation^22^, with fascaplysin did not reduce STAT3-induced migration (Supplementary Fig. 3k, l). However, inhibiting the activity of ROCK or myosin II suppressed STAT3-mediated migration (Fig. 3j and Supplementary Fig. 3m), suggesting that STAT3-mediated amoeboid movement is not attributed to increased cell proliferation. Next, we investigated the crosstalk between STAT3 and the major signal for amoeboid movement, i.e., Rho-ROCK signaling, in DLBCL. STAT3 phosphorylation was not affected by the ROCK inhibitor. In contrast, suppressing STAT3 activity or knocking down STAT3 reduced RhoA activity (Fig. 3k–m); in addition, overexpressing wild-type STAT3 increased phosphorylated MLC (Supplementary Fig. 3n), suggesting that STAT3 is located upstream of Rho-ROCK in DLBCL. Collectively, these results suggest that in DLBCL, JAK-STAT3 functions upstream of the Rho-ROCK pathway to regulate amoeboid movement.

### The IL-10-JAK-STAT3 loop contributes to DLBCL motility

**Fig. 4:**
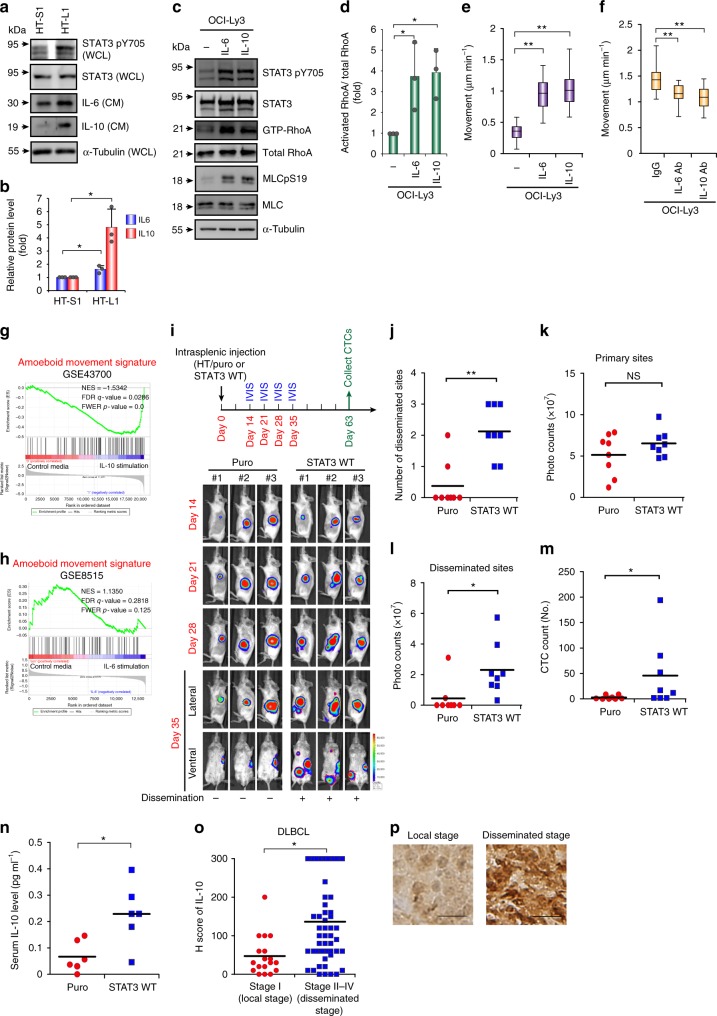
The IL10-JAK-STAT3 positive feedback loop contributes to DLBCL motility. **a**, **b** Representative results of western blots of indicated proteins in whole-cell lysates (WCL), IL-6 and IL-10 in conditioned media (CM) from HT-S1 and HT-L1 sublines. Quantification of IL-6 and IL-10 expression. *n* = 3. Data represent mean ± S.D. **c**, **d** OCI-Ly3 cells were grown in collagen gels and treated with IL-6 or IL-10. Representative western blots and pull down assay for showing the level of indicated proteins. Quantification of RhoA activity. *n* = 3. Data represent mean ± S.D. **e** Quantification of movement speeds of OCI-Ly3 cells treated with IL-6 or IL-10 (*n* = 30). **f** Quantification of movement speeds of OCI-Ly3 cells treated with IgG, IL-6, or IL-10 neutralizing antibody (*n* = 30). **g** GSEA for showing the association between the signature of amoeboid movement and gene expression array from healthy peripheral blood mononuclear cells stimulated with IL-10 or control media (GSE43700). **h** GSEA for showing the association between the signature of amoeboid movement and gene expression array from monocyte-derived macrophages stimulated with IL-6 or control media (GSE8515). **i** HT cells expressing WT STAT3 or a control vector (puro) were injected into the spleen of SCID/beige mice. Mice bioluminescent signals on indicated days were detected (*n* = 8; other 5 mice in Supplementary Fig. 4i). **j** Quantification of disseminated numbers in **i** and Supplementary Fig. 4i. **k-l** Quantification of bioluminescent intensity of **i** and Supplementary Fig. 4i. from the primary sites (yellow dotted lines circled area) and the disseminated sites (red dotted lines circled area). NS, not significant. **m** Quantification of the circulating tumor cells (CTCs) collected from the mice described in panel i. *n* = 8. **n** HT cells expressing WT STAT3 or a control vector (puro) were intraperitoneal injected into SCID*/*beige mice for 9 weeks. The dot plot shows the serum concentrations of IL*-*10. *n* = 6. **o** Distribution of the H score for IL-10 expression from 73 DLBCL patients. **p** Representative results of immunohistochemical staining for IL-10. Scale bar = 20 μm.*, *P* < 0.05; **, *P* < 0.005 by Student’s *t*-test; Kolmogorov–Smirnov tests for GSEA. See also Supplementary Fig. 4

Because STAT3 is located upstream of RhoA and induces amoeboid movement, we examined whether the IL-6/IL-10-STAT3 positive feedback loop was involved in DLBCL migration. Overexpressing WT STAT3 increased IL-6 and IL-10 expression without significantly affecting IL-12 expression (Supplementary Fig. 4a, b). The metastatic subline HT-L1 expressed higher levels of IL-6 and IL-10 than the primary tumor subline HT-S1 (Fig. 4a, b). IL-6/IL-10 stimulation enhanced RhoA activity and cell motility (Fig. 4c–e and Supplementary Fig. 4c-e). This movement was reduced in DLBCL cells upon treatment with anti-IL-6 or anti-IL-10 antibodies (Fig. 4f and Supplementary Fig. 4f). The IL-10- but not IL-6-stimulated gene expression signature was associated with amoeboid movement (Fig. 4g, h), indicating that IL-10 may be the major upstream cytokine that regulates STAT3-coordinated signals for DLBCL movement. Next, we determined the impact of STAT3 overexpression on DLBCL spread in vivo. HT cells that stably expressed WT STAT3 or a control vector (Supplementary Fig. 4g, h) were injected into the spleens of mice. STAT3 overexpression in HT cells encouraged DLBCL cell dissemination and increased the number of CTCs without significantly affecting primary tumor growth (Fig. 4i–m and Supplementary Fig. 4i). Serum IL-10 levels were higher in mice receiving an intraperitoneal injection of HT-STAT3 WT than in mice in the control group (Fig. 4n). In the DLBCL patient samples, increased IL-10 expression was observed in the disseminated stages (stages II–IV) (Fig. 4o, p), especially in the ABC-DLBCL patient samples (Supplementary Fig. 4j). These results indicate that the IL-10-JAK-STAT3 positive feedback loop drives DLBCL motility to promote dissemination.

### STAT3 activates *RHOH* transcription to increase RhoA activity

We next investigated the mechanism of STAT3-induced cell movement in DLBCL. We showed that in DLBCL, STAT3 was located upstream of RhoA and induced DLBCL movement (Fig. 3k–m). We next investigated how STAT3 activated RhoA in DLBCL. Because STAT3 does not affect total RhoA expression (Fig. 3k, m), we first examined whether STAT3 induced the expression of Rho-guanine nucleotide exchange factors (RhoGEFs) to activate RhoA. Although certain RhoA signal-related genes were upregulated by STAT3 in DLBCL cells as detected by PCR array screening (Supplementary Fig. 5a), these genes were not consistently increased in the HT-L1 and HT-L2 metastatic sublines (Supplementary Fig. 5b). Then, we determined whether STAT3 activates the expression of other Rho family proteins because certain Rho family proteins are expressed in B lymphocytes and can regulate each other’s activity^23^. We screened the mRNA levels of Rho family members in DLBCL cell lines. The SCC cell line FaDu was used as a control. An increase in *RHOH* levels was observed in DLBCL cells (Supplementary Fig. 5c). The mRNA levels of *RHOH* were consistently upregulated in the cDNA microarray data from HT-L1 and HT-L2 cells (Supplementary Fig. 5d). RhoH is an atypical GTPase protein that lacks GTPase activity and is expressed mainly in hematopoietic cells^24^. However, its role in DLBCL is unclear. A public data analysis showed that upregulating RhoH expression correlated with a worse prognosis for DLBCL patients (Supplementary Fig. 5e).

**Fig. 5:**
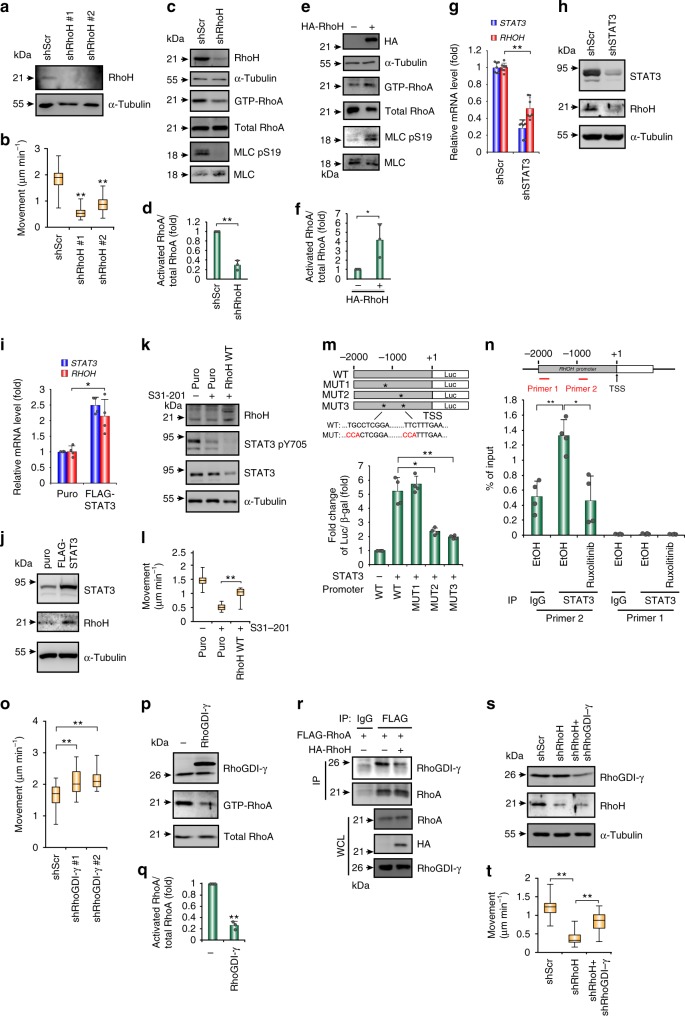
STAT3 induces *RHOH* transcription to activate RhoA through competitive interaction with RhoGDIγ. **a** Western blots of RhoH in SU-DHL-5 cells receiving shRNAs specific to RhoH (shRhoH; clones #1, #2) or a scramble sequence (shScr). **b** Quantification of movement speeds (*n* = 30). **c**, **d** Western blots for the indicated proteins and pull down assay for GTP-bound RhoA in SU-DHL-5 cells receiving shRhoH or shScr (**c**). Quantification of RhoA activity (*n* = 3) (**d**). **e**, **f** Pull down assay showing the GTP-RhoA in HT cells transfected with HA-RhoH or a control vector (**e**). Quantification of RhoA activity (*n* = 3) (**f**). **g**, **h** Analysis of the mRNA (**g**) (*n* = 7) and protein (**h**) expression of STAT3 and RhoH in SU-DHL-5 cells receiving shRNAs against STAT3 or a scramble sequence. **i, j** Analysis of the mRNA (**i**) (*n* = 4) and protein (**j**) expression of STAT3 and RhoH in HT cells stably expressed FLAG-STAT3 WT or control vector. **k** Western blots for showing the efficacy of inhibitors and the expression level of RhoH in SU-DHL-5 cells expressing HA-RhoH or control vector. **l** Quantification of the movement speeds (*n* = 20). **m** Luciferase reporter assay. The result is presented as luciferase activity/β-galactosidase (β-gal). *n* = 4. A schema for the promoter region of the *RHOH* gene and reporter constructs. *mutated sites. **n** ChIP assay in SU-DHL-5 after ruxolitinib treatment for 24 h. Primer 1, control. Primer 2, amplifying fragment containing STAT3-binding element (−992–−983, TTCTTTGAA). *n* = 4. **o** Quantification of movement speeds of SU-DHL-5 cells receiving shRNAs specific to RhoGDIγ (shRhoGDI-γ; clones #1, #2) or shScr. *n* = 30. **p**, **q** Western blots and the pull down assay for GTP-bound RhoA in HA-RhoGDI-γ or control vector transfected HT cells (**p**). Quantification of RhoA activity (*n* = 3) (**q**). **r** Immunoprecipitation-western blot of HT cells stably expressed FLAG-RhoA transfected with indicated plasmids. **s** Western blots of RhoGDIγ and RhoH in HT cells receiving shRNAs against RhoH, RhoGDIγ, or a scramble sequence. **t** Quantification of movement speeds (*n* = 30). Data represent mean ± S.D. in panels **d**, **f**, **g**, **i**, **m**, **n**, and **q**. *P < 0.05, **P < 0.005 by Student’s *t*-test. See also Supplementary Fig. 5 and Supplementary Movie 4

We next examined whether RhoH participated in STAT3-induced RhoA activation and amoeboid movement. RhoH knockdown suppressed cell movement (Fig. 5a, b, Supplementary Fig. 5f and Supplementary Movie 4) and reduced RhoA activity (Fig. 5c, d), and ectopically expressed RhoH activated RhoA (Fig. 5e, f). The suppression of STAT3 expression by shRNAs decreased RhoH mRNA and protein levels (Fig. 5g, h), and ectopically expressed STAT3 upregulated RhoH (Fig. 5i, j). Overexpressing RhoH partially rescued the inhibition of cell migration mediated by STAT3 suppression (Fig. 5k, l). We further determined whether STAT3 directly activated *RHOH* transcription. STAT3 activated the proximal promoter of *RHOH*, and mutations to the STAT3*-*binding sites abrogated this activation (Fig. 5m). Chromatin immunoprecipitation confirmed the direct binding of STAT3 to the *RHOH* promoter, and suppressing STAT3 transcriptional activity with ruxolitinib diminished this binding (Fig. 5n).

Next, we determined how RhoH regulated RhoA activity to control amoeboid movement in DLBCL. Among the major regulators of Rho GTPases, i.e., RhoGEFs, Rho GTPase-activating proteins (RhoGAPs), and Rho GDP-dissociation inhibitors (RhoGDIs), RhoH has been shown to interact with RhoGDIs^24^. We hypothesized that RhoH interacted competitively with RhoGDIs to activate RhoA in DLBCL. We first examined the role of RhoGDIs in DLBCL movement. Of the three RhoGDIs, RhoGDIγ was predominantly expressed in SU-DHL-5 cells (Supplementary Fig. 5g). Depleting RhoGDIγ increased amoeboid movement (Fig. 5o and Supplementary Fig. 5h). The ectopic expression of RhoGDIγ in HT cells reduced GTP-bound RhoA and MLC S19 phosphorylation (Fig. 5p, q and Supplementary Fig. 5i), suggesting that RhoGDIγ may play a negative role in amoeboid movement by deactivating RhoA. We further examined whether RhoH interacted with RhoGDI to sequester it and activate RhoA. Among the three RhoGDIs, RhoGDIγ apparently interacted with RhoA, and ectopically expressed RhoH disrupted this interaction (Fig. 5r and Supplementary Fig. 5j). A dose-dependent effect of RhoH on competing for RhoA–RhoGDIγ interaction was demonstrated (Supplementary Fig. 5k-l). RhoH interacted with RhoGDIγ (Supplementary Fig. 5m). RhoGDIγ knockdown compensated for the reduction in amoeboid movement caused by RhoH depletion (Fig. 5s, t). Collectively, these data suggest that in DLBCL, STAT3 transactivates *RHOH*, resulting in RhoA activation through the sequestration of RhoGDIγ by RhoH to alleviate RhoGDI-mediated RhoA inactivation.

### STAT3 suppresses tubulin acetylation and activates RhoA

**Fig. 6:**
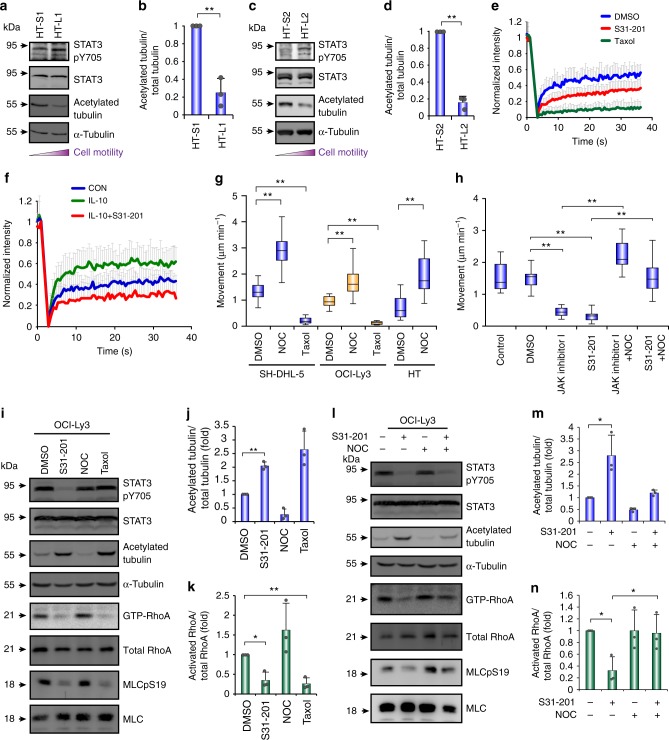
STAT3 activates RhoA through regulating microtubule dynamics. **a**, **c** Western blots for showing the level of Y705-phosphorylated STAT3, total STAT3, and acetylated tubulin in HT-L1 vs. HT-S1 (**a**) and HT-L2 vs. HT-S2 (**c**) sublines. **b**, **d** Quantification of relative level of acetylated tubulin in **a** and **c**, respectively (*n* = 3). **e**, **f** Fluorescence recovery after photobleaching (FRAP) of STAT3 WT-expressed HT cells stably expressing cherry-tubulin. The inhibitors were added 4 h before FRAP (**e**). FRAP of HT-STAT3 cells, IL-10-treated HT-STAT3 cells, and IL-10-treated HT-STAT3 cells with S31-201 which stably expressed cherry-tubulin. The cells were grown in collagen gels in serum-free medium and then treated with IL-10 (**f**). Quantification of fluorescence intensity before and after bleaching. The graph illustrates fluorescence recovery curves. (*n* ≥ 25). **g** Quantification of movement speeds of SU-DHL-5, OCI-Ly3, or HT cells treated with different inhibitors (*n* ≥ 20). **h** Quantification of movement speeds of SU-DHL-5 cells treated with different inhibitors (*n* = 20). **i**, **l** Representative results of western blots and pull down assay for showing the level of Y705-phosphorylated STAT3, acetylated tubulin, and active (GTP-bound) Rho family proteins in OCI-Ly3 cells under the treatment of different inhibitors for 6 h. **j**, **m** Quantification of relative level of acetylated tubulin in **i** and **l**, respectively (*n* = 3). **k**, **n** Quantification of RhoA activity in **i** and **l**, respectively (*n* = 3). Data represent mean ± S.D. in panels **b**, **d**, **e**, **f**, **j**, **k**, **m**, and **n**. *P < 0.05, **P < 0.005 by Student’s *t*-test. See also Supplementary Fig. 6 and Supplementary Movie 5. See Supplementary Table 4 for the working concentrations of inhibitors

We investigated whether there were other mechanisms of STAT3-regulated DLBCL movement in addition to RhoH activation. RhoH knockdown did not suppress DLBCL movement to the level observed when STAT3 or ROCK were inhibited (Supplementary Fig. 6a), and amoeboid movement in DLBCL cells was impaired 6 h after STAT3 inhibitor treatment (Fig. 3d). Therefore, we assumed that the transactivation of *RHOH* by STAT3 was not the only pathway for STAT3-mediated amoeboid movement and that STAT3 may act in a transcription-independent manner to induce DLBCL motility. Tyrosine 705-unphosphorylated STAT3 enhances microtubule polymerization and tubulin acetylation by sequestering the tubulin-binding protein stathmin 1^25,26^. We determined whether STAT3 activation affected microtubule polymerization, as well as its impact on RhoA activation. The HT-L1 and HT-L2 metastatic sublines displayed higher levels of STAT3 Y705 phosphorylation and lower levels of acetylated tubulin than the HT-S1 and HT-S2 primary tumor sublines (Fig. 6a–d). There was a trend for an inverse association between STAT3 activity, as well as cell motility, and acetylated tubulin levels in the DLBCL cell lines (Supplementary Fig. 6b, c). Inhibiting STAT3 activity increased the acetylated tubulin levels (Supplementary Fig. 6d). Furthermore, suppressing STAT3 activity reduced the microtubule dynamics as detected by fluorescence recovery after photobleaching (FRAP) (Fig. 6e and Supplementary Fig. 6e). IL-10 stimulation increased the microtubule dynamics, and suppressing STAT3 activity in IL-10-treated cells reduced these effects (Fig. 6f). Disrupting microtubules with nocodazole (NOC) facilitated amoeboid movement. Conversely, enhancing microtubule formation with Taxol impaired movement (Fig. 6g, Supplementary Fig. 6f and Supplementary Movie 5). Moreover, NOC treatment restored JAK/STAT3 inhibition-suppressed motility (Fig. 6h), suggesting that microtubules play a negative role in the amoeboid movement of DLBCL cells and are located downstream of STAT3. There was a trend that activated RhoA inversely correlated with acetylated tubulin levels in response to treatment with S31-201, NOC, and Taxol (Fig. 6i–k and Supplementary Fig. 6g-i). Disrupting microtubules with NOC compensated for the STAT3 inhibition-mediated suppression of RhoA activity (Fig. 6l–n and Supplementary Fig. 6j-l).

We next investigated the mechanism through which STAT3 activated RhoA by regulating microtubule dynamics. Microtubules have been reported to increase RhoA activity by sequestering ARHGEF2, which enhances cell motility^27^. We examined whether microtubules sequester ARHGEF2 to increase the migration of DLBCL cells. Inhibition of STAT3 activity by S31-201 increased the fraction of insoluble microtubules (Supplementary Fig. 6m). Moreover, ARHGEF2 knockdown impaired amoeboid movement (Supplementary Fig. 6n, o), suggesting that the activation of STAT3 regulates microtubule dynamics to control the amoeboid movement of DLBCL by releasing ARHGEF2.

### Exploration of strategies for attenuating DLBCL progression

**Fig. 7:**
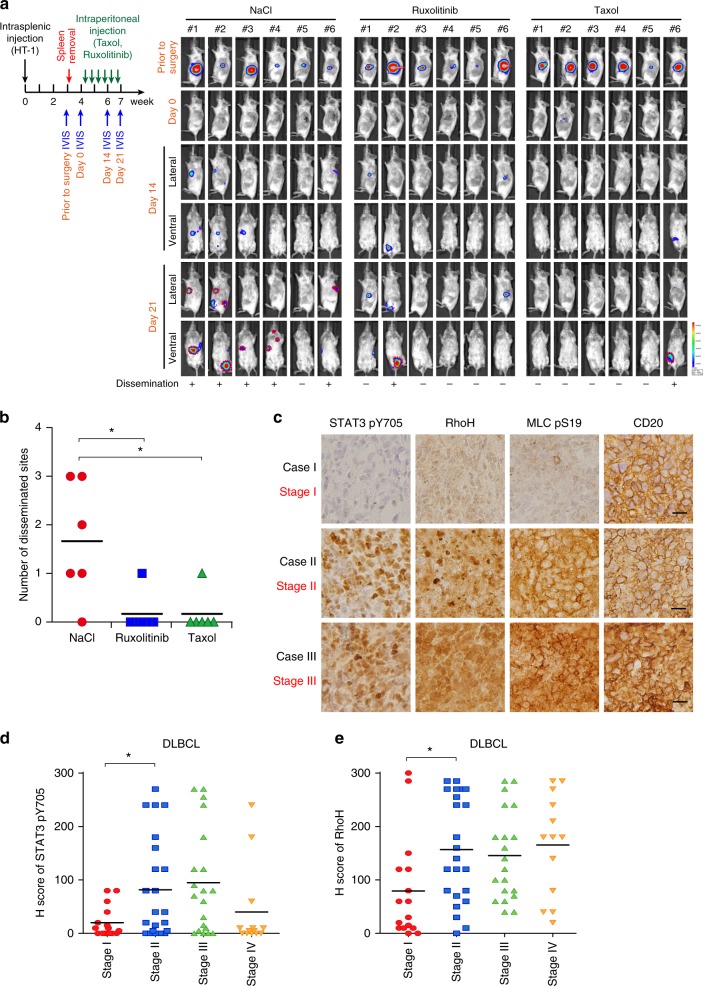
Validation of the JAK/STAT3-microtubule-RhoA axis in DLBCL in vivo and clinical samples. **a** A schema for showing the experimental design of the mice experiment. The HT-1 subline was injected into the spleen of SCID/beige mice. Before Taxol or Ruxolitinib injection, the primary tumors were removed. The drugs were injected intraperitoneally on Day 0 and bioluminescent signals were taken at the indicated time points. NaCl, vehicle control. *n* = 6 for each group. **b** Quantification of disseminated lesions (red dotted lines) in **a**. **c** Representative pictures of immunohistochemical staining of the Y705-phosphorylated STAT3, and S19-phosphorylated MCL, RhoH, and CD20 in DLBCL cases. The clinical stage of the representative cases is indicated. Scale bar = 25 μm. **d** Distribution of H scores of Y705-phosphorylated STAT3 in DLBCL patients with different stages. **e** Distribution of H scores of RhoH in DLBCL patients with different stages. *P < 0.05 by Student’s *t*-test. See also Supplementary Fig. 7

Next, we examined the impact of JAK/STAT3-RhoA, as well as microtubules on DLBCL progression in vivo. To further exclude the effects of primary tumor growth on tumor dissemination, the tumorigenic HT-1 subline (see Fig. 2a) was injected into the spleens of mice, and the primary sites (spleen) were removed before treatment. Ruxolitinib and Taxol treatment significantly impaired DLBCL dissemination in vivo (Fig. 7a, b). Finally, we validated the proposed mechanism in DLBCL patient samples. Representative IHC scoring results are shown in Supplementary Fig. 7a, and images of representative cases with different stages are presented in Fig. 7c. Correlations between the levels of RhoH and phosphorylated STAT3, the levels of RhoH and phosphorylated MLC, and the levels of phosphorylated STAT3 and phosphorylated MLC were noted in DLBCL patients (Supplementary Fig. 7b-d). Importantly, these correlations were validated in ABC-DLBCL patients (Supplementary Fig. 7 e-g). Disseminated DLBCL (stage II–IV) had increased STAT3 phosphorylation, RhoH expression, and MLC phosphorylation (Fig. 7d, e and Table 1). STAT3 phosphorylation and RhoH expression were also noted to be higher in advanced-stage ABC-DLBCL cases than in stage I cases (Supplementary Fig. 7h, i).

**Table 1:** A table for showing the mean value ± S.D. of H score of the indicated markers in local (stage I) vs. disseminated (stage II–IV) DLBCL patients

	Stage I (Local stage)	Stage II–IV (Disseminated stage)	*P* value
MLC pS19	89.69 ± 92.23	155.30 ± 104.2	0.013
RhoH	79.38 ± 95.62	154.73 ± 91.30	0.0026
STAT3 pY705	20 ± 28.81	76.60 ± 92.95	0.0097

**Fig. 8:**
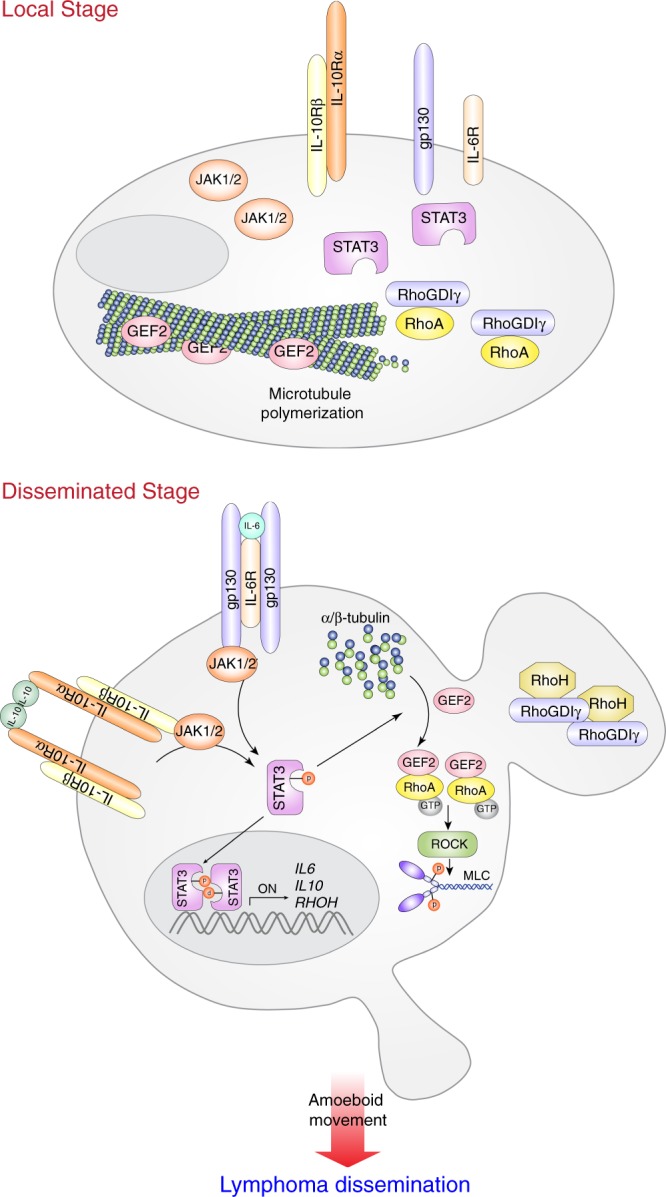
A schema for showing the molecular mechanism guiding cell movement in disseminated stage DLBCL

Here, we summarize the findings shown in Fig. 8. In localized DLBCL, unphosphorylated STAT3 permits microtubule formation to sequester ARHGEF2 (GEF2). In addition, RhoGDIγ interacts with RhoA to suppress its activity. In disseminated DLBCL, IL-6/IL-10 induces STAT3 activation. Phosphorylated STAT3 induces RhoH expression, which releases RhoA from RhoGDIγ. Furthermore, activated STAT3 causes microtubule disruption, leading to ARHGEF2 (GEF2) release to activate RhoA. All of these events coordinately promote the dissemination of DLBCL.

## Discussion

The distinct outcomes of local stage and disseminated stage DLBCL patients indicate a change in the basic character of the tumor cells during the dissemination/progression of DLBCL^28^. However, the current understanding of DLBCL migration is relatively limited compared with the abundant knowledge regarding lymphomagenesis and tumor growth. In this study, we showed that STAT3 coordinates different signals to regulate DLBCL migration, which contributes to the early dissemination and progression of DLBCL. Furthermore, we demonstrated the proliferation-independent effect of STAT3 in driving DLBCL migration and dissemination. An intriguing issue is that in contrast to most studies that consider STAT3 as a pro-survival and proliferative factor^29^, our study sheds new insight into the role of STAT3 in DLBCL motility beyond cellular proliferation. Importantly, the clinical sample analysis strongly supports the existence of the proposed signaling mechanism in DLBCL cases, especially in ABC-type DLBCL, which harbors constitutive NF-κB activation^30^. Previous studies indicate that NF-κB induces cytokines, such as IL-6/IL-10, to maintain oncogenic signaling in DLBCL^9,31^. Here, we showed that IL-6/IL-10 contributes to DLBCL migration through activating STAT3-driven migratory mechanisms. Whether NF-κB participates in DLBCL dissemination and whether inhibiting NF-κB activity attenuates DLBCL progression deserve further investigation.

In this study, we have extensively validated the crucial role of RhoA activation in DLBCL migration. Regarding genomic alterations, RhoA mutations have been reported in lymphomas. The *RHOA* Gly17Val inactivating mutation has been noted in angioimmunoblastic T-cell lymphoma^32^. In B-cell lymphoma, inactivating mutations of *GNA13*, a gene encoding Gα_13_ that is an upstream activator of RhoA, are more commonly found in Burkitt’s lymphoma^33,34^. However, the current knowledge is not sufficient to support the role of RhoA mutations as a driving event in lymphomas. Instead, mutations to epigenetic regulators, including EZH2, MLL2, CREBBP, and EP300, have been shown to act as drivers or accelerators in DLBCL^35–38^. We suggest that RhoA activation is the critical event in late-stage progression rather than a driving event in lymphomagenesis. Intercepting this signaling pathway will be of great importance in combating advanced DLBCL. The exact role of *RHOA* mutations in lymphomagenesis requires additional clarifying evidence.

A major clinical implication of this study is the consideration of different treatment strategies in early and disseminated DLBCL. The current gold standard for DLBCL treatment is the “R-CHOP” regimen, regardless of the patient stage^39^. Microtubule stabilizers and JAK inhibitors are not included in this regimen. Here, we show that the microtubule-stabilizing agent Taxol and the JAK1/2 inhibitor ruxolitinib effectively suppress the dissemination of DLBCL by impeding STAT3-driven migratory pathways in DLBCL. Ruxolitinib is used primarily for myeloproliferative neoplasms^40^, and its application in DLBCL is limited. Reports of Taxol use in B-cell lymphoma are also limited; primarily, it is used to treat refractory diseases^41^. According to our report of the distinct characters and activated signals of localized versus disseminated DLBCLs, considering stage-specific treatments for DLBCL may further improve the treatment outcomes of advanced-stage patients.

In conclusion, our study identified the previously overlooked characteristics and underlying mechanism of DLBCL motility. An understanding of the mechanism underlying lymphoma cell migration not only explains the distinct clinical characteristics between patients with DLBCL and solid tumors but also has a considerable impact on the development of therapies tailored to patients’ specific DLBCL clinical stage.

## Methods

### Human samples and DLBCL subtype determination

Seventy-three patients with pathologically confirmed DLBCL diagnosed within the past decade at Chang-Gung Memorial Hospital Chiayi were enrolled in the study. Only immunocompetent patients with adequate paraffin-embedded biopsy specimens collected at the time of diagnosis were included. Information regarding a variety of characteristics, including patient demographics, clinical stage, therapy type, treatment outcomes, and survival status, was obtained and is shown in Supplementary Table 1. Risk stratification was conducted using the International Prognostic Index (IPI) score. From the 73 DLBCL patients, 40 available patient samples were subjected to an 8-gene expression panel analysis to determine the GCB and ABC subtypes as reported previously^42–44^. RNA was extracted from FFPE tissue (ReliaPrep™ FFPE Total RNA Miniprep System, Promega) and used for cDNA synthesis. For GCB confirmation, *LMO2*, *MYBL1*, *BCL6*, and *NEK6* expression levels were increased in the RNA samples. The ABC subtype was indicated by relatively higher expression of *IRF4*, *FOXP1*, *IGHM*, and *TNFRSF13B*. The gene expression was normalized to an internal control (*ACTB*), and the fold change was calculated by dividing the average dCT of each gene. This study was approved by the Institutional Review Board of Chang-Gung Memorial Hospital (104-2442B).

### Cell lines

DLBCL cell lines (SU-DHL-5, OCI-Ly3, HT, DB, and U2932) and human head and neck cancer cell lines (OEC-M1 and FaDu) were grown in RPMI medium supplemented with 10% FBS, penicillin-streptomycin, L-glutamine, and sodium pyruvate. HEK293T cells were grown in DMEM supplemented with 10% FBS and penicillin-streptomycin. DB, FaDu, and HEK293T cells were purchased from the Bioresource Collections and Research Center of Taiwan. OCI-Ly3 and U2932 cells (originally purchased from Deutsche Sammlung von Mikroorganismen und Zellkulturen (DSMZ), Germany) were gifts from Dr. Chun-Yu Liu (Taipei Veterans General Hospital, Taiwan). SU-DHL-5 and HT cell lines were obtained from Dr. Kung-Chao Chang (National Cheng Kung University, Taiwan) and have been described previously^45^. The OEC-M1 human head and neck cancer cell line was provided by Dr. Kuo-Wei Chang (National Yang-Ming University of Taiwan). All the cell lines used in our studies have been tested for Mycoplasma contamination and authenticated by STR method.

### Mouse experiments

All procedures were conducted in accordance with the institutional animal welfare guidelines of Taipei Veterans General Hospital. CB17.Cg-*Prkdc*^*scid*^*Lyst*^*bg-J*^/CrlBltw (SCID/beige) mice were purchased from BioLASCO Taiwan Co., Ltd. For the orthotopic xenograft mouse model, cells were injected into the splenic parenchymas of 7- to 8-week-old SCID/beige mice (Figs. 2b, 2e, 2g, 4i, 4n, 7a and Supplementary Fig. 2d, 2h, 4i). For the drug treatment experiment shown in Fig. 7a, the primary tumors were removed 3 weeks after injection, and the mice were then injected intraperitoneally with 10 mg/kg Taxol, 30 mg/kg ruxolitinib, or 0.9% NaCl twice a week for an additional seven weeks. The bioluminescence signals in the mice were detected each week using a Caliper IVIS Spectrum System (Caliper Life Sciences, Inc., Waltham, MA). HT cells expressing FLAG-tagged STAT3 WT or the control vector were injected intraperitoneally into SCID/beige mice to detect the serum levels of IL-10. After 9 weeks, the mice were sacrificed; serum samples were collected, and IL-10 levels were measured using a Human IL-10 High Sensitivity ELISA kit (BMS215HS, eBioscience, Inc., San Diego, CA) (Fig. 4n). For the detection of circulating tumor cells (CTCs) in Figs. 2k and 4m, samples were prepared and analyzed using the MiSelect R system. The murine blood samples were transferred from K 2 EDTA tubes into correspondingly labeled 50-ml conical centrifuge tubes. The samples were incubated with PE-conjugated anti-CD19 for 20 min at room temperature. After staining, Isoton diluent was added to each tube. The samples were centrifuged at 800× *g* for a full 10 min with the brake off in a swing bucket centrifuge at room temperature. Following centrifugation, the supernatants were removed, and the samples were mixed for the CTC analysis. The samples were processed on the MiSelect R System within 1 h of the sample preparation. A cell was classified as a CTC when its morphological features were consistent with those of a tumor cell and when it was positive for CD19, GFP, and DAPI staining. The CTC number was counted and analyzed by the operators and recorded directly. The animal experiments were approved by the Institutional Animal Care and Utilization Committee of National Yang-Ming University (IACUC No. 2014-086).

### Immunohistochemistry

For immunohistochemistry, deparaffinization with xylene, antigen retrieval with 10 mM sodium citrate, permeabilization with Triton, and antibody hybridization and visualization were performed according to the Novolink™ Polymer Detection System (Leica Biosystems) manufacturer’s instructions. For immunohistochemical grading, immunoscores were defined as follows: 0, no staining; 1, very weak staining; 2, weak staining; and 3, strong staining (Supplementary Fig. 7a). The H score was calculated by multiplying the immunoscore value by the percentage of the positive area.

### Recombinant DNA and reagents

The pCMV-HA-RhoH expression vector was generated by inserting full-length cDNAs (RhoH: NM_001278369) into the pCMV-HA vector. The pFLAG-CMV2-STAT3 WT, mutant (Y705F and Y604F), and pFLAG-CMV2-RhoGDIα/β/γ expression vectors were generated by inserting the full-length cDNAs (STAT3: NM_139276; RhoGDIα: NM_001185077; RhoGDIβ: NM_001175; and RhoGDIγ: NM_001176) into pFLAG-CMV2 vectors. The pEBG-RhoH expression vectors were generated by inserting full-length cDNAs into the pEBG vector. The RhoH promoter region (−2000 to +1 of TSS) was cloned into the pGL4.2 vector. The pcDNA3-STAT3 WT vector was generated by inserting the full-length cDNA into a pcDNA3 vector. The pCDH-puro-FLAG-STAT3 WT and mutant (Y705F, Y604F, and E434/435A) vectors were generated by inserting the appropriate full-length FLAG-STAT3 sequences into the pCDH-puro vector. The pLAS1w.3xLacO-shSTAT3 vectors were generated by inserting the shScr or shSTAT3 oligonucleotides (Supplementary Table 6) into the pLAS1w.3xLacO vector. Recombinant human IL-6, IL-10, and IL-12 were purchased from PeproTech (Rock Hill, NJ). Recombinant human His-tagged RhoA was purchased from Cytoskeleton Inc. (Denver, CO).

### Virus production and infection

pCMV-Δ8.9, pMDG (from the National RNAi Core Facility, Taiwan), and lentiviral expression vectors (pCDH-GFP, pCDH-puro, pLKO.1-shsScr, and shRNA clones) were co-transfected into HEK293T cells overnight to package the virus. Then, the medium was replaced, and the virus-containing supernatant was collected 48 and 72 h after transfection. For virus transduction, the cells were mixed with virus-containing supernatants supplemented with 8 μg/ml polybrene (Sigma-Aldrich, St. Louis, MO), and the mixture was centrifuged at 1000× *g* for 90 min at 37 °C. After infection with the virus for 24 h, the infected cells were selected with 1 μg/ml puromycin for three days.

### Immunofluorescence and morphological analyses of cells

Lymphoma cells were grown on 1.5 mg/ml collagen gel, and SCC cells were grown on 1.5 mg/ml collagen gel or plastic culture dishes overnight. The cells were fixed in 4% paraformaldehyde in phosphate-buffered saline (PBS) for 1 h at room temperature and permeabilized with 0.1% Triton X-100 in PBS for 30 min at room temperature. The fixed cells were stained with primary antibodies overnight at 4 °C and then incubated overnight at 4 °C with Alexa-Fluor-488-conjugated secondary antibodies (Invitrogen Carlsbad, CA). The primary antibodies used for immunofluorescent staining are listed in Supplementary Table 5. Chemifluorescence signals were detected using a fluorescence imaging system (Olympus FluoView™ FV1000 confocal microscope; Olympus Corporation, Tokyo, Japan). Cell morphology was described and is represented as the ratio of the perimeter^2^ to the 4π area. Images were captured using a Leica DM IRBE microscope (Leica Microsystems, Wetzlar, Germany), and the areas and perimeters of the cells (*n* = 50) were determined using ImageJ software.

### Fluorescence recovery after photobleaching

HT cells or STAT3-expressing HT cells were stably transfected with cherry-tubulin. The cells were grown in 3D collagen gel overnight and then treated with inhibitors for 4 h or cytokines for 24 h. Pre-bleached images were captured before bleaching. The cells (*n* ≥ 25 for each condition) were bleached for 3 s in a 2 μm × 2 μm square area, and fluorescence images were captured every 0.5 s for 35 s. The fluorescence intensity was normalized to that of the reference region. The recovery intensities were subtracted from the bleach intensities and then normalized to the pre-bleach intensity.

### Trajectory tracking of DLBCL cell migration

Lymphoma cells were suspended overnight in 200 μl of a 1.5 mg/ml collagen solution (Advanced BioMatrix, Inc. Carlsbad, CA). The cells were observed for 6 h in a humidified, CO_2_-equilibrated chamber with a Leica DM IRBE microscope equipped with a motorized stage (Leica Microsystems, Wetzlar, Germany). Images were captured every 5 min for 6 h. For the inhibitor experiments, inhibitors were added 1 h before tracking. For antibody neutralization, antibodies were added the day before tracking. For cytokine stimulation, the cells were grown in serum-free media for 24 h, and cytokines (100 ng/ml IL-6 or IL-10) were added the day before tracking. The movement of cells in each group was tracked for 73 frames, and each frame was taken every 5 min. In randomly selected high-power fields (0.65 mm^2^), we selected cells at random to represent the movement of the majority of the population (>75%) using trajectory tracking with ImageJ software. In each independent experiment, 10 cells were counted, and the results were displayed with Box-and-whisker plots. The cells were not counted when they were out of focus or disappeared in the captured fields during the tracking duration. Vertical migration was not counted in our system. Cell motility speed was calculated and is presented as micrometers per minute. Due to the difficulty in identifying cell boundaries by algorithm, cell edges were traced manually using ImageJ. The center of mass (COM) of the individual cells was then determined for tracking the COM trajectory **r**(*t*_*i*_), where **r** = (r_*x*_, r_*y*_) is the COM position, and *t*_i_ is the time of frame i. For each cell, we calculated the COM displacement (*d* = sqrt[(**r**(*t*_*i*_)−**r**(*t*_*i*-1_))^2^]) after each frame. Thus, the measurements of a single cell trajectory produced 73 displacements. All displacements from the same cell line were used to plot the histograms. The total number of samples was 73 × 30 (cell number). The probability of displacement was calculated by dividing the number of occurrences by the total number of samples.

### Immunoblotting and immunoprecipitation

The cells were lysed in 1% Nonidet P-40 lysis buffer (1% Nonidet P-40, 50 mM Tris-HCl, pH 7.4, 150 mM NaCl, 5% glycerol, and 1 mM Na_3_VO_4_ plus protease inhibitors (Roche, Mannheim, Germany)) and incubated on ice for 30 min. Next, the cell lysates were centrifuged at 17,000× *g* for 10 min, and the supernatants were collected. For microtubule fractionation, the cells were lysed in 0.5% Triton lysis buffer (0.5% Triton, 50 mM Tris-HCl, pH 7.4, and 150 mM NaCl plus protease inhibitors (Roche, Mannheim, Germany)) and incubated at RT for 5 min. Next, the cell lysates were centrifuged at 17, 000x *g* for 60 min; then, we collected the supernatants as the Triton-soluble fractions and the pellets as the Triton-insoluble fractions. The protein concentrations were determined with BCA protein assays (Thermo Scientific Pierce™ BCA Protein Assay, Waltham, MA) and an Infinite M200 microplate reader (Tecan, Switzerland). To disrupt the protein structure, 2× sample buffer was added to each sample and mixed. The mixtures were heated at 95 °C for 10 min. Then, the denatured proteins were loaded on 6%–12% SDS-PAGE gels for separation with running buffer. The proteins were then transferred onto PVDF membranes from Millipore (Billerica, MA) at 300 mA on ice for 2 h. The membranes containing the denatured proteins were blocked with 5% skim milk in TBST at room temperature for 1 h. Then, the membranes were incubated with the specific primary antibodies (Supplementary Table 5) at 4 °C overnight. The membranes were washed with TBST and incubated with secondary antibodies in 5% skim milk for 1 h at room temperature. The membranes were washed in TBST again and then incubated with ECL from Millipore (Billerica, MA). The results were analyzed using a GE LAS-4000 (GE Healthcare Inc., Marlborough, MA). To collect conditioned media, 2 × 10^6^ cells were grown in 2 ml of serum-free media for 24 h. The proteins in the conditioned media were precipitated using trichloroacetic acid and then analyzed by immunoblotting. For immunoprecipitation, the lysates were mixed with an equal amount of 1% Nonidet P-40 lysis buffer (1% Nonidet P-40, 50 mM Tris-HCl, pH 7.4, 150 mM NaCl, 5% glycerol and 1 mM Na_3_VO_4_ plus protease inhibitors (Roche, Mannheim, Germany)). Primary antibodies or IgG was added to the lysates; the samples were incubated at 4 °C on a rotary device for 2 h and then mixed with Protein A/G beads (Invitrogen, Carlsbad, CA) for an additional 2 h. The beads were collected by centrifugation and washed gently with 1% Nonidet P-40 lysis buffer three times before immunoblotting. For the competition assay, purified GST-RhoH and RhoA were incubated with immobilized FLAG-RhoGDI-γ for 2 h. The primary antibodies used for immunoblotting and immunoprecipitation are listed in Supplementary Table 5. Chemiluminescence signals were detected and quantified using a luminescence imaging system (LAS-4000, Fujifilm Holdings Corporation, Minato, Tokyo). The blots were presented as cropped with at least one marker for indicating the molecular weight position. The uncropped scans of all blots are shown in Supplementary Figure 8 of Supplementary Information.

### Quantitative RT-PCR (RT-qPCR) and RhoA signaling PCR array

RNA was extracted using Trizol, and the total amount of RNA was quantified using a Nanodrop spectrophotometer (Thermo Fisher Scientific). One microgram of RNA was used for reverse-transcription with the OneStep RT-PCR Premix Kit (Bionovas, Taiwan) according to the manufacturer’s instructions. For qPCR, the reaction mixtures (20 µl) contained 10 µl of SYBR Green (Thermo Fisher Scientific), 0.5 µM forward and reverse primers, and 0.2 µl of cDNA. After an initial denaturation cycle (95 °C for 20 s), the product was amplified at 95 °C for 3 s and 60 °C for 30 s for 40 PCR cycles using an Applied Biosystems real-time quantitative PCR instrument (Thermo Fisher Scientific). The Human RHOA Pathway TaqMan® Array (4418825, Thermo Fisher Scientific, Inc., Waltham, MA), which contains 92 genes associated with the RhoA pathway, was used to identify the genes that were differentially expressed in the DB cells compared with the SU-DHL-5 cells and in the HT cells expressing the control vector (puro) compared with the HT cells expressing WT STAT3. The PCR array was performed according to the manufacturer’s instructions.

### Small GTPase activity assay

For small GTPase activity assays, GTP-bound RhoA in whole-cell lysates was pulled down by the immobilized GST-Rhotekin Rho-binding domain. GTP-bound Rac1 and Cdc42 in whole-cell lysates were pulled down by the immobilized GST-p21-activated kinase Rac binding domain. The washed complexes were analyzed by immunoblotting with antibodies specific for RhoA, Cdc42, and Rac1.

### Chromatin immunoprecipitation

For chromatin immunoprecipitation (ChIP) assays, chromosomal DNA fragments were prepared according to the manufacturer’s instructions (Thermo Scientific Pierce™ Magnetic ChIP Kit, Waltham, MA). Briefly, the lysates were incubated with 2 μg IgG or a STAT3-specific antibody.

### Reporter assay

The Gemini X2 System (BTX, Fisher Scientific, Pittsburgh, PA) was used for transient transfections. For the reporter assays, 2 μg of full-length or mutated reporter constructs, 3 μg of a pCMV-β-gal internal control plasmid and 40 μg of pFLAG-CMV2-STAT3 WT were co-transfected into HT cells (7 × 10^6^ cells) using the Gemini X2 System (BTX) and incubated for 48 h before luciferase activity was measured.

### cDNA microarray analysis

The gene expression patterns of the HT sublines derived from primary sites (spleens; Sx, S1, and S2) and disseminated sites (lymph nodes; Lx, L1, and L2), were analyzed using the Affymetrix Human Genome U133 Plus 2.0 Array (Thermo Fisher Scientific, Inc., Waltham, MA). Biotinylated cRNAs were prepared from 200 ng of total RNA using the GeneChip® 3′ IVT PLUS Reagent Kit according to the standard Affymetrix protocol, and the GeneChips were scanned with the GeneChip® Scanner 3000 7 G using the standard Affymetrix protocol. The gene ontology analysis was performed with DAVID Bioinformatics Resources 6.7 (http://david.abcc.ncifcrf.gov/).

### GST-fusion protein purification

GST-tagged RhoH was expressed in *E*. *coli* DH5α following induction with isopropyl-d-thiogalactopyranoside (IPTG). The bacteria were lysed in lysis buffer (1% Triton in PBS), and the GST-tagged proteins were immobilized on glutathione*-*Sepharose beads. The complexes were washed once with lysis buffer and then eluted with elution buffer (10 mM reduced glutathione in 50 mM Tris, pH 8.0).

### Gene set enrichment analysis

The microarray gene expression datasets from DLBCL patients (GSE11318)^46^, IL-10-stimulated peripheral blood mononuclear cells (GSE43700)^47^, and IL-6-stimulated human monocyte-derived macrophages (GSE8515)^48^ were downloaded from the National Center for Biotechnology Information website. Two public databases (GSE11318 and GSE43700) were generated by Affymetrix GPL570 (Affymetrix Human Genome U133 Plus 2.0 Array), and the public database GSE8515 was generated by Affymetrix GPL96 (Affymetrix Human Genome U133A Array, Santa Clara, CA). Gene Set Enrichment Analysis (GSEA) was performed with GSEA software from the Broad Institute. The correlation of the defined set of genes (shown in Supplementary Data 2) with a phenotype was considered statistically significant. The signature genes from different database for GSEA analysis were referred to the original articles^19,49–54^. NES, normalized enrichment score; FDR, false discovery rate; FWER: family-wise error rate.

### Statistics

Two-tailed, independent Student’s *t*-tests were used to compare continuous variables between two groups. Chi-squared tests were used to compare dichotomous variables. Kolmogorov–Smirnov tests were used for GSEA. The Kaplan–Meier method and log-rank tests were used to compare survival between patient groups. All statistical data were derived from at least two independent biological replicates, and each experiment contained at least two technical replicates. The numerical results are presented as the mean ± S.D. Box-and-whisker plots show the distribution of the data: maximum (upper end of the whisker), upper quartile (top of the box), median (band in the box), lower quartile (bottom of the box), and sample minimum (lower end of the whisker). The horizontal lines in the dot plots represent the mean value of each group. The level of statistical significance was set to *P* ≤ 0.05 for all of the tests.

## Electronic supplementary material

Supplementary Information

Description of Additional Supplementary Files

Supplementary Data 1

Supplementary Data 2

Supplementary Movie 1

Supplementary Movie 2

Supplementary Movie 3

Supplementary Movie 4

Supplementary Movie 5

## Data Availability

All relevant data are available from the corresponding author upon reasonable request. The datasets obtained from the cDNA microarray of the HT cells and the derived sublines (HT-Sx, HT-Lx, HT-S1, HT-L1, HT-S2, and HT-L2) were deposited in the Gene Expression Omnibus (GEO) database under accession number GSE81968. The URL for Supplementary Fig. 5e which analyzed the DLBCL survival from a publically available dataset is http://dna00.bio.kyutech.ac.jp/PrognoScan-cgi/PrognoScan.cgi?DATA_POSTPROCESSING=None&PROBE_ID=2004478&TITLE=Prognostic+value%20of%20RHOH%20mRNA%20expression%20in%20Blood%20cancer&TEST_NUM=23&MODE=SHOW_GRAPH.
