# Supplementary material for: STAT3-coordinated migration facilitates the dissemination of diffuse large B-cell lymphomas
Source: Nat Commun. 2018 Sep 12;9:3696. doi: 10.1038/s41467-018-06134-z (PMC6135800; doi:10.1038/s41467-018-06134-z)
Supplement: Supplementary file 1 — Supplementary Information [file 41467_2018_6134_MOESM1_ESM.pdf]

Supplementary information

STAT3-coordinated migration facilitates dissemination of diffuse large B-cell lymphomas

Pan et al.

Supplementary Figures

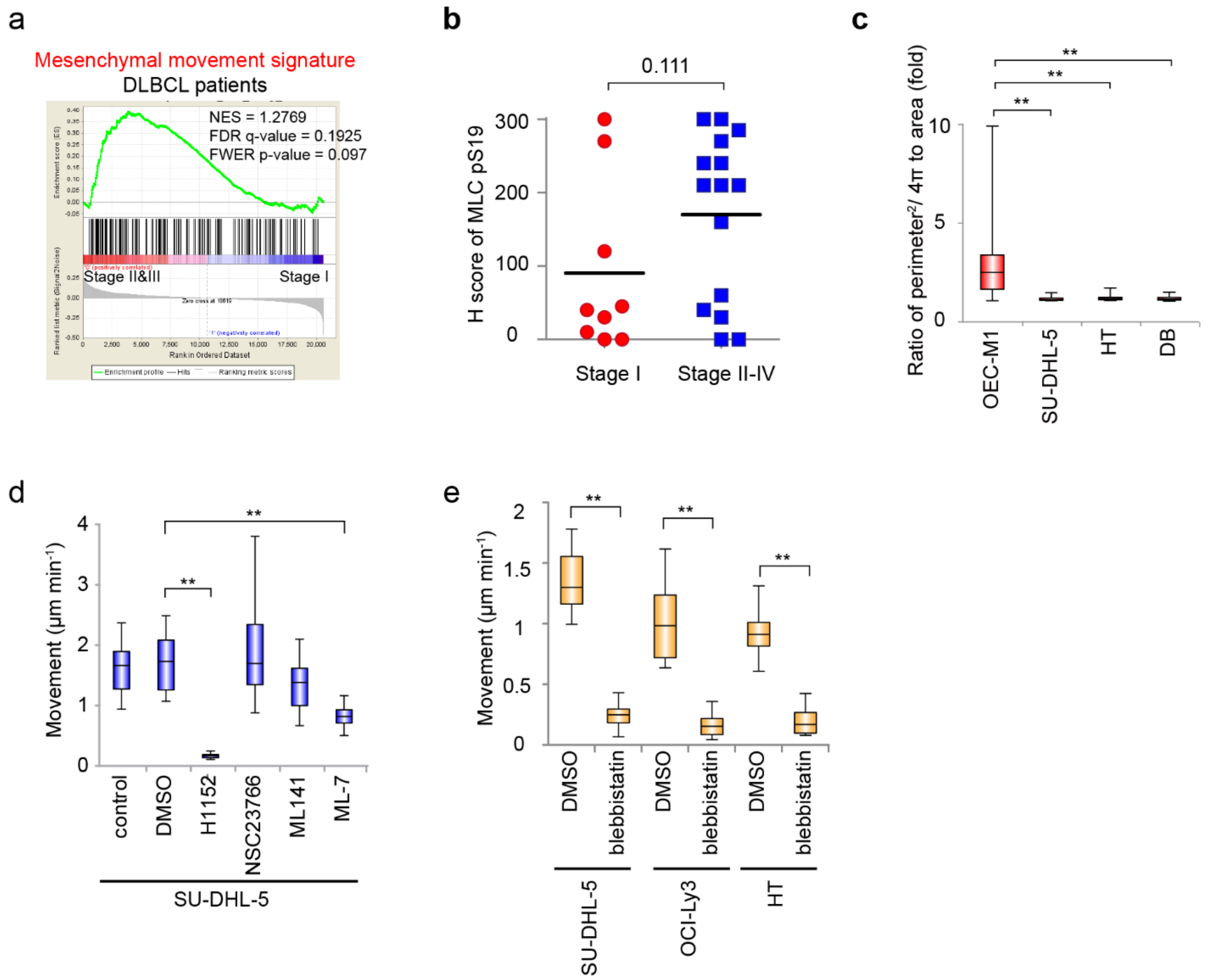

Supplementary Fig. 1 Amoeboid movement is detected in DLBCL in vitro. **a** GSEA for examining the association between the mesenchymal movement signature (Supplementary Data 2) and signature in DLBCL patients with stage II-III vs. stage I (GSE11318). **b** Distribution of the H score for MLC S19 phosphorylation (MLCpS19) from 24 ABC-type DLBCL patients. **c** Quantification of cellular morphology. SU-DHL-5, HT and DB cells were grown in collagen gels, and OEC-M1 cells were grown on collagen gels overnight. The images of cellular morphology were obtained by phase contrast microscopy. n=50. **d** Quantification of movement speeds of SU-DHL-5 cells treated with inhibitors (n=20). **e** Quantification of movement speeds of SU-DHL-5 cells treated with blebbistatin (n=20).

**, $P < 0.005$ by Student's t -test; Kolmogorov-Smirnov tests for GSEA.

See Supplementary Table 2 for chi-squared test for MLC S19 phosphorylation; Supplementary Table 4 for working concentration of inhibitors.

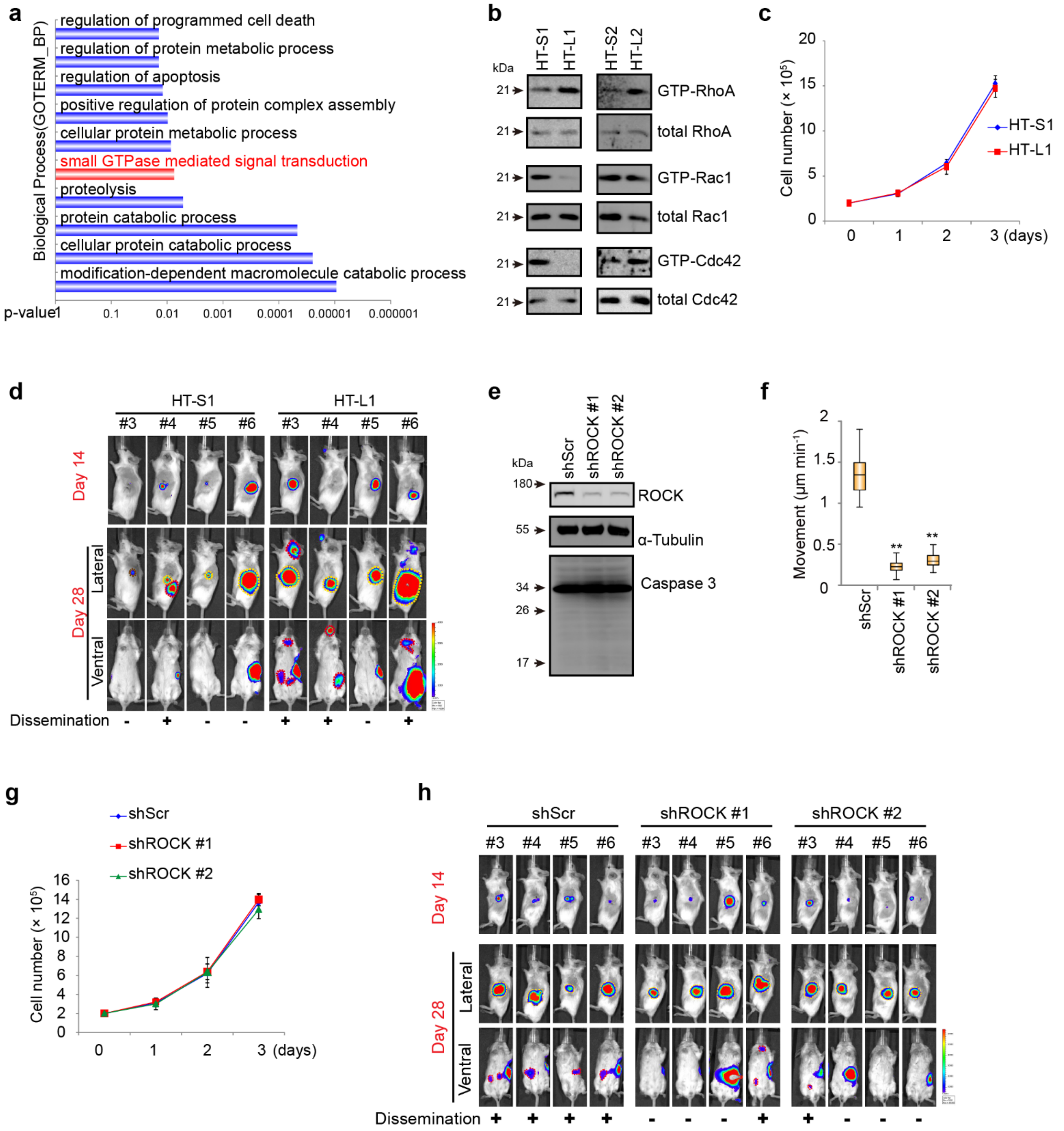

Supplementary Fig. 2 Amoeboid movement is detected in DLBCL in vivo. **a** Gene ontology analysis for showing the major activated signals in motile sublines HT-L1/HT-L2 compared with HT-S1/HT-S2. **b** Western blots and pull down assay for showing the level of active RhoA, total RhoA, active Rac1, total Rac1, active Cdc42 and total Cdc42 in HT-S1, HT-S2, HT-L1, and HT-L2 sublines **c** Cell proliferation assay. 2×10^4 HT-S1 and HT-L1 cells were grown in 6 well. The cell number was counted every 24 hours after seeding for 3 days (n=6 for each cell line). Data represent mean ± S.D. **d** Investigation of the in vivo dissemination of S1 and L1 sublines. The cells were injected into the splenic parenchyma. Mice bioluminescent signals on indicated days were detected (n= 6; other 2 mice in Fig. 2e) **e** Western blots of ROCK and caspase 3 in HT-L1 subline receiving shRNAs specific to ROCK I (shROCK; clones #1, #2) or scramble (shScr). **f** Quantification of movement speeds of the cells described in panel e (n=30). **g** Cell proliferation assay. 2×10^4 HT-L1 receiving shRNAs specific to

ROCK I (shROCK; clones #1, #2) or a scramble sequence (shScr) were grown in 6 well. The cell number was counted every 24 hours after seeding for 3 days (n=6 for each cell line). Data represent mean ± S.D. **h** Investigation of the in vivo dissemination of HT-L1 receiving shRNAs specific to ROCK I (shROCK; clones #1, #2) or a scramble sequence (shScr). The cells were injected into the splenic parenchyma. Mice bioluminescent signals on indicated days were detected (n= 6; other 2 mice in Fig. 2g)

**, $P < 0.005$ by Student's t -test.

See Supplementary Data 1 for cDNA microarray analysis; Supplementary Table 4 for working concentration of inhibitors.

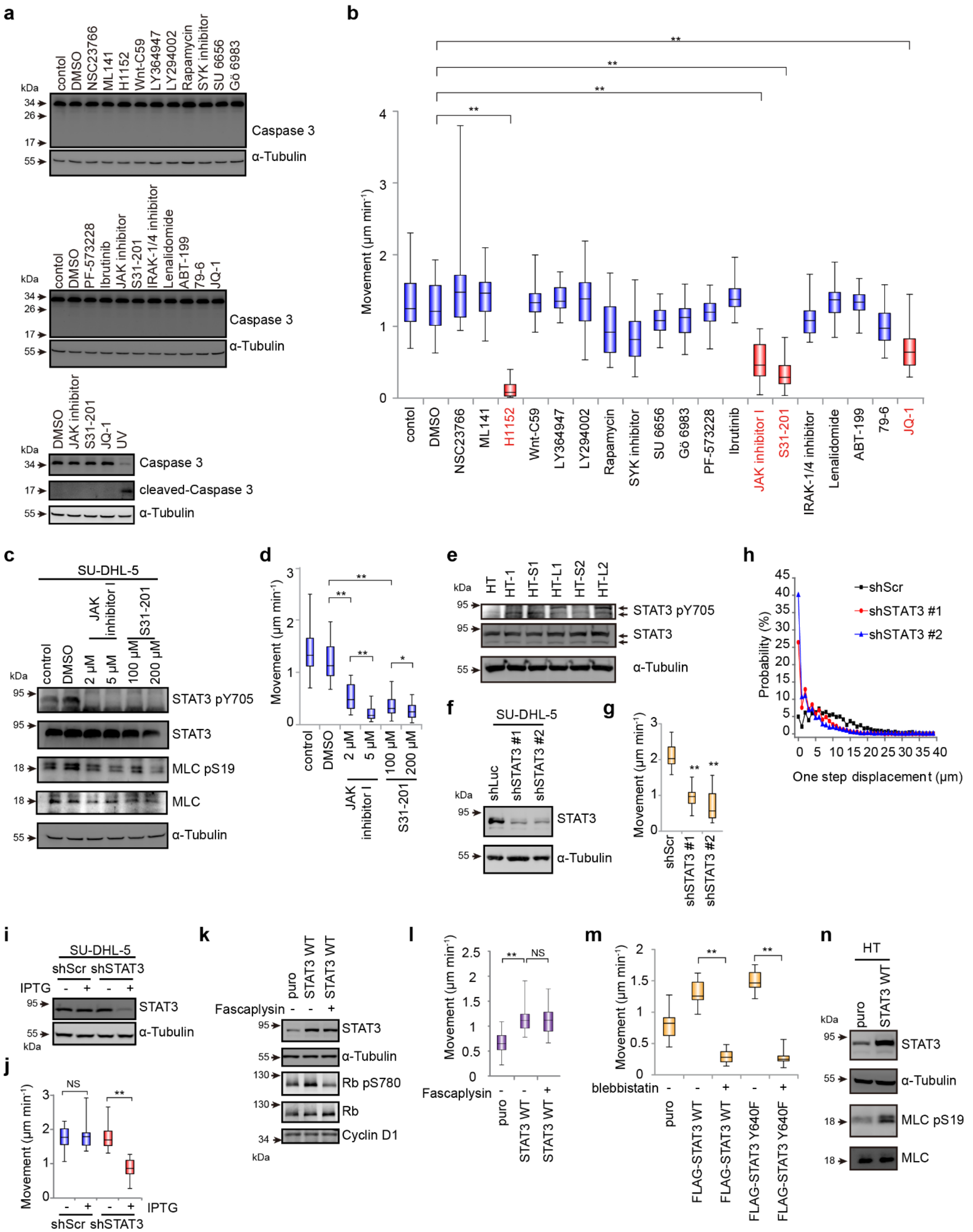

Supplementary Fig. 3 JAK-STAT3 signaling mediates amoeboid movement via RhoA activation in DLBCL. **a** SU-DHL-5 cells were grown in collagen gels overnight and then treated with inhibitors for 6 hours. Western blots for showing full length or cleaved Caspase 3. α-tubulin was a loading control. **b** Quantification of movement speeds of inhibitor treated SU-DHL-5 cells (n=40). **c** Western blots of indicated proteins from inhibitor-treated SU-DHL-5 cells. **d** Quantification of movement speeds of inhibitor-treated SU-DHL-5 cells (n=40) **e** Western blots for showing STAT3, STAT3 Y705

phosphorylation and tubulin in HT and HT- derived sublines. **f** Western blots for showing the knockdown efficacy of SU-HL-5 cells receiving shRNAs specific to STAT3 (shSTAT3; clones #1, #2) or scramble (shScr). **g** Quantification of movement speeds of the cells described in panel **f** (n=30). **h** The probability of one-step displacement of the cells described in panel **f.** **i** Western blots for showing the knockdown efficacy of SU-DHL-5 cells receiving LacO/IPTG inducible shRNAs specific to STAT3 (shSTAT3) or a scramble sequence (shScr). The cells were incubated in the presence (+) or absence (-) of 1 mM IPTG for 5 days. **j** Quantification of movement speeds of the cells described in panel i (n=30). NS, Not significant. **k** Western blots for showing the inhibitor efficacy of HT cells expressing STAT3 WT or control vector (puro) treated fascaplysin or DMSO. **l** Quantification of movement speeds of the cells described in panel k (n=30). NS, Not significant. **m** Quantification of movement speeds of HT cells expressing STAT3 WT, Y640F or control vector (puro) treated blebbistatin or DMSO (n=20). NS, Not significant. **n** Western blots of indicated proteins from HT cells expressing STAT3 WT or control vector (puro).

*, P < 0.05; **, P < 0.005 by Student's t -test.

See Supplementary Table 4 for the working concentration of the inhibitors.

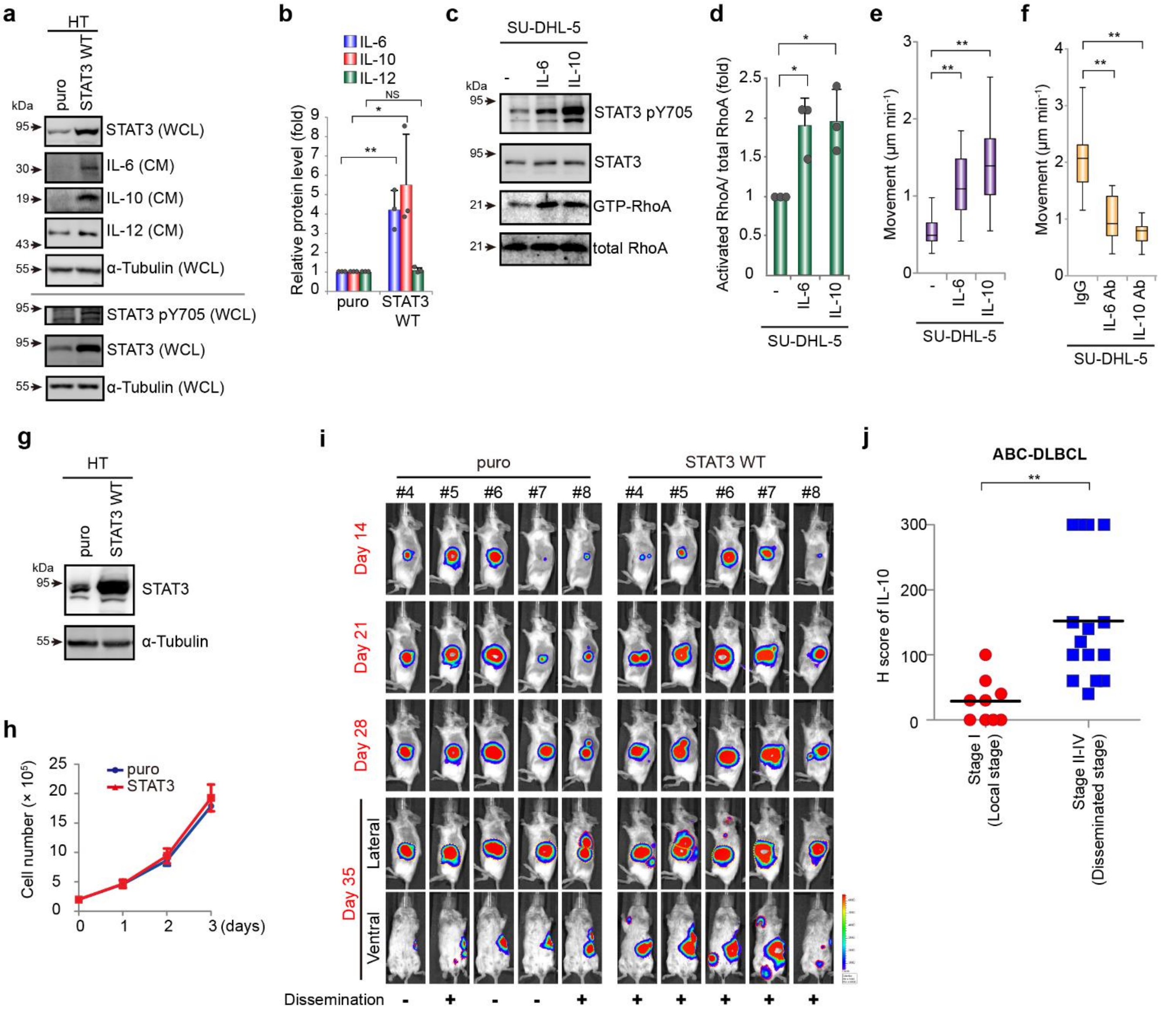

Supplementary Fig. 4 The IL10-JAK-STAT3 positive feedback loop contributes to DLBCL motility in vitro and in vivo. **a-b** Representative results of STAT3 pY705 and total STAT3 in whole cell lysates (WCL) and IL-6, IL-10, IL-12 in conditioned media (CM) of FLAG-STAT3 WT or control vector (puro) stably expressed HT cells (**a**). Quantification of IL-6 and IL-10 expression from three independent experiments (**b**). CM were harvest after 24 h culture. Data represent mean ± S.D. **c-d** Representative results of STAT3 pY705, total STAT3, RhoA, and GTP-RhoA (by pull down assay) in SU-DHL-5 cells grown in collagen gels and treated with IL-6 or IL-10 (**c**). Quantification of RhoA activity from three independent experiments. Data represent mean ± S.D (**d**). **e** Quantification of movement speeds of SU-DHL-5 cells treated with IL-6 or IL-10 (n=20). **f** Quantification of movement speeds of SU-DHL-5 cells treated with IgG, IL-6 or IL-10 neutralizing antibody (n=20). **g** Western blots for showing the level of total STAT3 in HT-Luc cells expressing FLAG-tagged STAT3 WT or control vector (puro). **h** Cell Proliferation assay. 2×10^4 HT cells expressing STAT3 WT or control vector (puro) were grown in 6 well. The cell number was counted every 24 hours after seeding for 3 days (n=12 for each cell line). Data represent mean ± S.D. **i** HT cells expressing WT STAT3 or a control vector (puro) were injected into the spleen of SCID/beige mice. Mice bioluminescent signals on indicated days were detected (n= 8; other 5 mice in Fig. 4i). **j** Distribution of the H score for IL-10 expression from 24 ABC-type DLBCL patients. *, $P < 0.05$; **, $P < 0.005$ by Student's t -test.

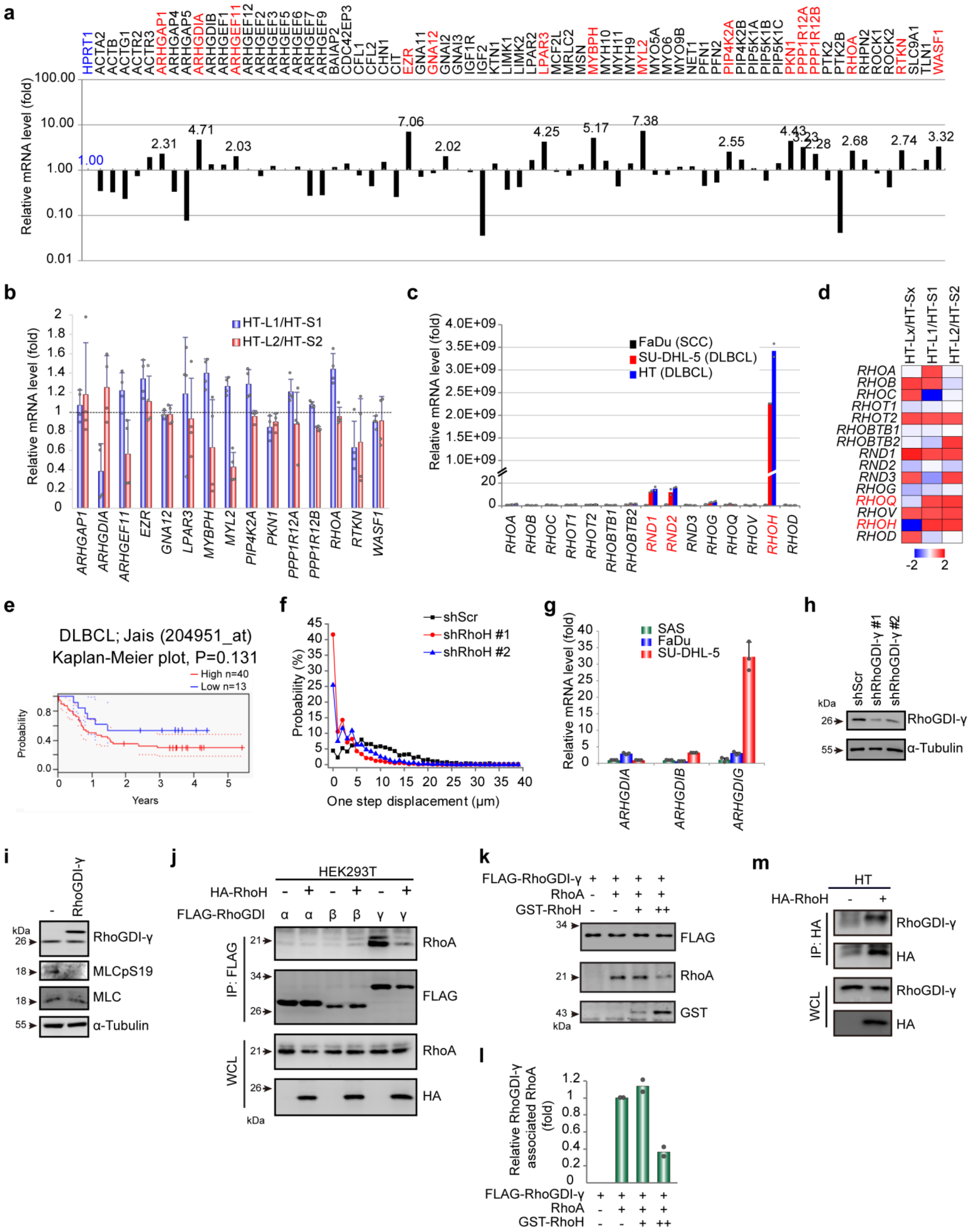

Supplementary Fig. 5 RhoH increases DLBCL motility and correlates with a worse prognosis in DLBCL. **a** The relative mRNA levels of RhoA-pathway related molecules analyzed by TaqMan® Array Human RHOA Pathway. Data were presented as fold changes relative to HT cells expressing control vector. Blue: the control gene. Red: the changes of the expression of genes in HT cells stably expressing FLAG-tagged STAT3 WT ≥ two folds. **b** RT-qPCR of HT-L1 or HT-L2 cells vs. HT-S1 or HT-S2 (n=4). **c** RT-qPCR of DLBCL cell lines (SU-DHL-5 and HT) and the SCC cell line (FaDu) (n=2). **d** A heat

map showing the differentially expressed genes of Rho family in the sublines isolated from primary sites (HT-Sx, HT-S1 and HT-S2) and from the disseminated sites (HT-Lx, HT-L1, HT-L2). Data were presented as the fold changes of the disseminated sublines vs. corresponding primary sublines. **e** Kaplan-Meier plot of DLBCL cases with high vs. low *RHOH* from public dataset (accession number: E-TABM-346). Dotted line, 95% confidence intervals. **f** The probability of one-step displacement of SU-DHL-5 cells receiving shRNA against RhoH or a scrambled sequence. **g** RT-qPCR of SU-DHL-5 and the SCC cell lines (SAS and FaDu) (n=3). **h** Western blots for showing the knockdown efficacy of SU-HL-5 cells receiving shRNAs specific to RhoGDIγ (shRhoGDI-γ; clones #1, #2) or scramble (shScr). **i** Western blots of indicated proteins from HT cells expressing RhoGDIγ WT or control vector (-). **j** Immunoprecipitation-western blots in HEK-293T cells transfected with the indicated plasmids. **k-l** Competition assay. FLAG-RhoGDIγ was transiently expressed in HEK293T cells and was immunoprecipitated. The immunocomplexes were subjected to a competition assay in the presence (+) or absence (−) of purified RhoA WT or GST-RhoH WT. The immunocomplexes were analyzed by immunoblotting. **k**, representative blots; **l,** quantification of relative level of RhoGDIγ-associated RhoA (n=2). Data represent mean. **m** Immunoprecipitation-western blots in HT cells transfected with the indicated plasmids.

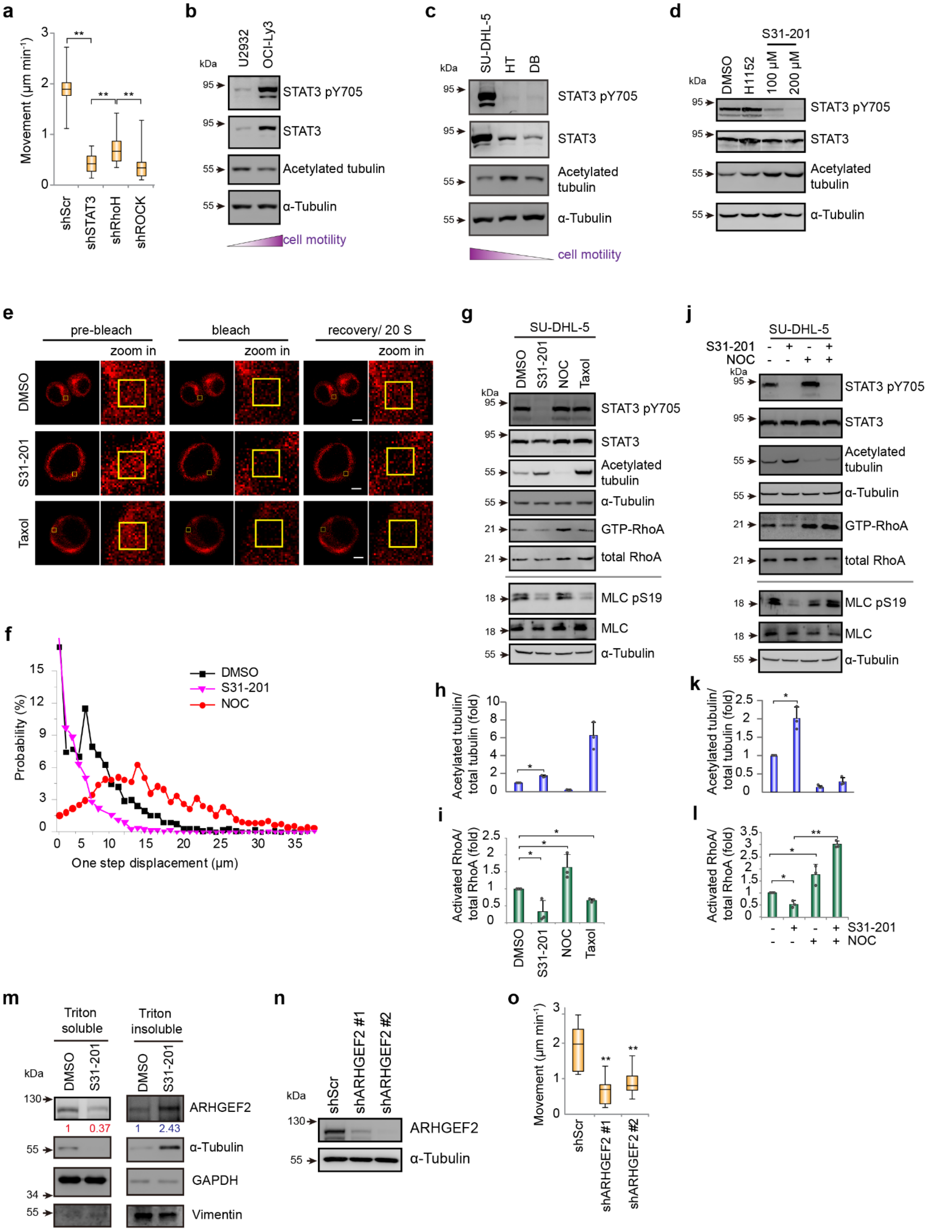

Supplementary Fig. 6 STAT3 activates RhoA through regulating microtubule dynamics to release ARHGEF2. **a** Quantification of the movement speed of the SU-DHL-5 cells receiving shRNAs against STAT3 (shSTAT), RhoH (shRhoH), ROCK I (shROCK), or scramble (shScr). n=30. **b,c** Western blots for showing the level of Y705-phosphorylated STAT3, total STAT3, acetylated tubulin and tubulin in

DLBCL cells with different motility. **d** Western blots for showing the level of Y705-phosphorylated STAT3, total STAT3, acetylated tubulin and tubulin in SU-DHL-5 treated H1152, S31-201 or DMSO. **e** STAT3 WT expressed HT cells expressing cherry-tubulin were grown in 3D collagen gels overnight and treated with S31-201, taxol or DMSO. Before bleaching, the images were caught (pre-bleach). The cells were bleached for 3 seconds in an area of 2 μm x 2 μm square (bleach) and fluorescence recovery was measured 20 seconds after bleaching (recovery). Yellow squares indicate bleaching areas. **f** The probability of one-step displacement of SU-DHL-5 cells treated with DMSO, S31-201, or nocodazole (NOC). **g,j** Representative results of western blots and pull down assay for showing the level of Y705-phosphorylated STAT3, acetylated tubulin, and active (GTP-bound) Rho family proteins in SU-DHL-5 cells under the treatment of different inhibitors for 6 hours**.** **h,k** Quantification of relative level of acetylated tubulin from panel **g** and **j**, respectively (n=3). Data represent mean ± S.D. **i,l** Quantification of RhoA activity from panel **g** and **j**, respectively (n=3). Data represent mean ± S.D. **m** Microtubule fractionation. Soluble tubulin and insoluble tubulin fractionation are separated by triton in DMSO or S31-201 (100 μM for 6 hr) treated SU-DHL-5 cells. GAPDH is the loading control for triton-soluble proteins, and vimentin is the loading control for triton-insoluble proteins. The different fractions were analyzed by immunoblotting with the indicated proteins. **n** Western blots for showing the knockdown efficiency in SU-DHL-5 cells receiving shRNAs specific to ARHGEF2 (shARHGEF2; clones #1 and #2) or scramble (shScr). **o** Quantification of movement speeds (n=20).

*, $P < 0.05$; **, $P < 0.005$ by Student's t -test.

See Supplementary Table 4 for the working concentration of the inhibitors.

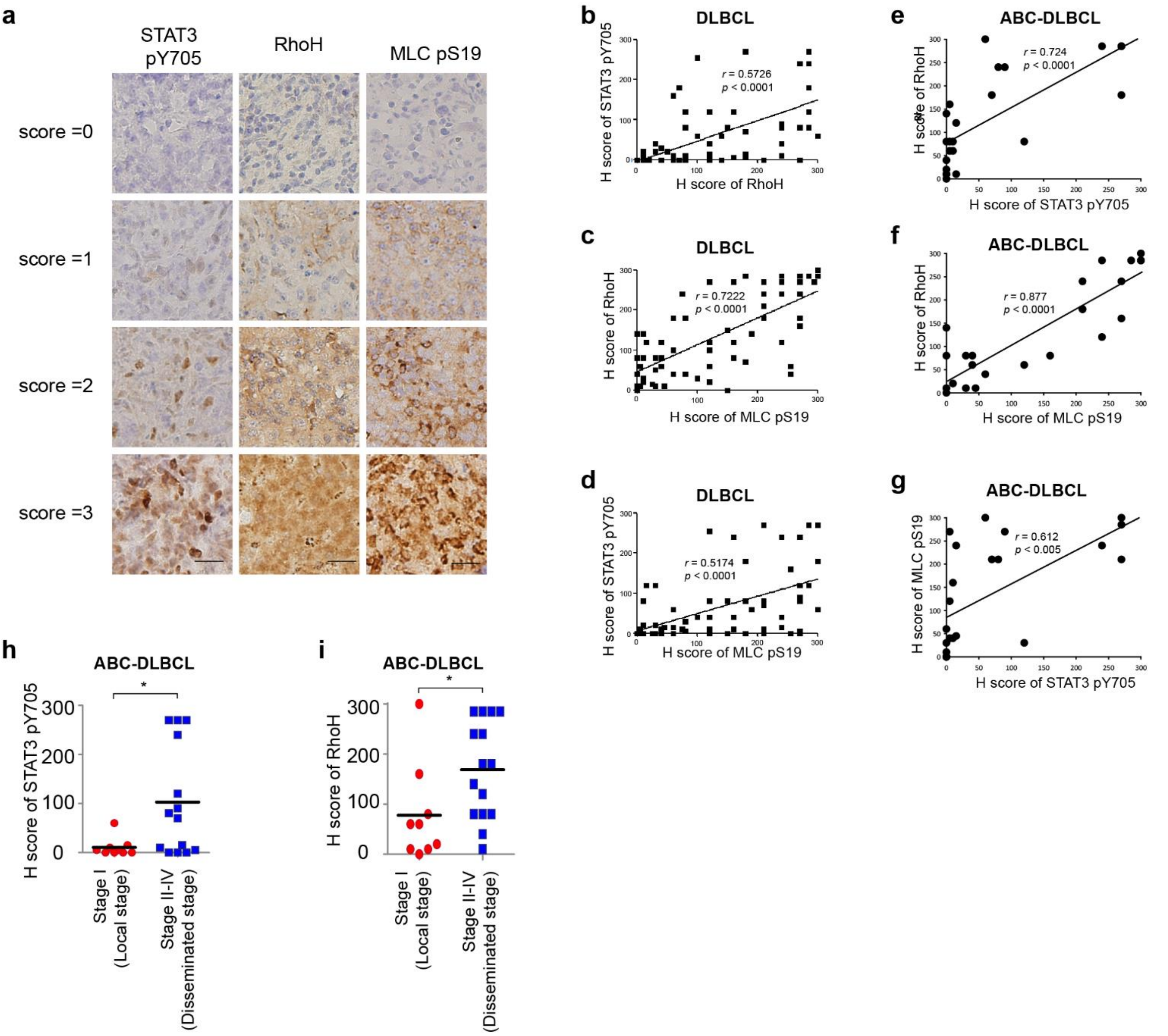

Supplementary Fig. 7 Validation of the proposed STAT3-driven mechanism in DLBCL in vivo and clinical samples. **a** Representative images for immunohistochemical scoring of S19-phosphorylated MLC, RhoH, and Y705-phosphorylated STAT3 in DLBCL patients. Scale bar=20 μm. **b-d** The correlation between the level of Y705-phosphorylated STAT3 (STAT3 pY705) and RhoH (**b**), S19-phosphorylated MLC II (MLC pS19) and RhoH (**c**), and STAT3 pY705 and MLC pS19 (**d**) in 72 DLBCL patients. The correlation coefficients *r* and P values are shown in each panel. **e-g** The correlation between the level of Y705-phosphorylated STAT3 (STAT3 pY705) and RhoH (**e**), S19-phosphorylated MLC II (MLC pS19) and RhoH (**f**), and STAT3 pY705 and MLC pS19 (**g**) in 24 ABC-type DLBCL patients. The correlation coefficients *r* and P values are shown in each panel. **h** Distribution of H scores of Y705-phosphorylated STAT3 in 24 ABC-type DLBCL patients with different stages. **i** Distribution of H scores of RhoH in 24 ABC-type DLBCL patients with different stages.

*, $P < 0.05$ by Student's t test.

Main Figures

Fig. 3d

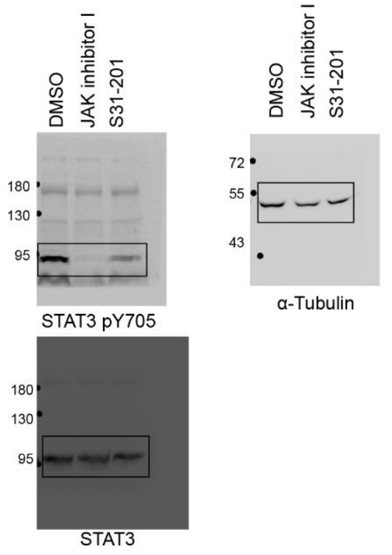

Fig. 3f

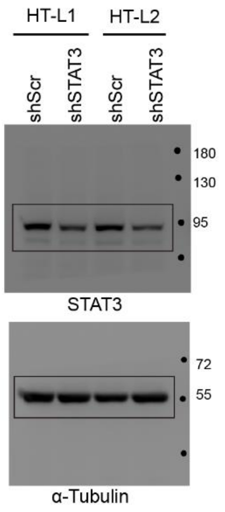

Fig. 3h

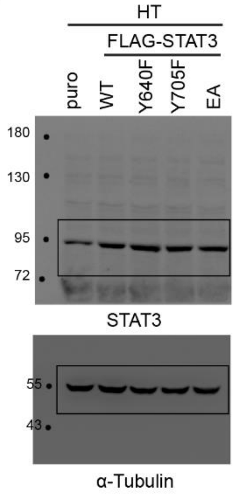

Fig. 3k

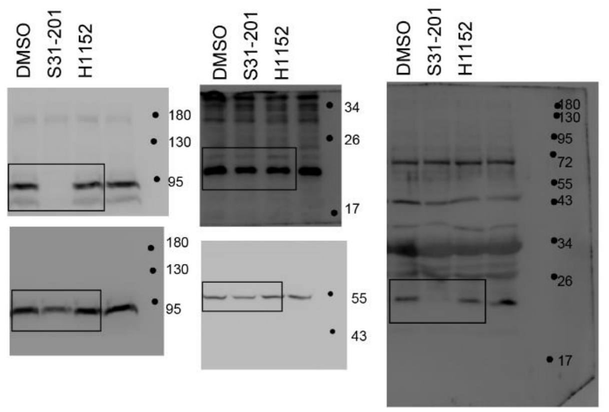

Fig. 3m

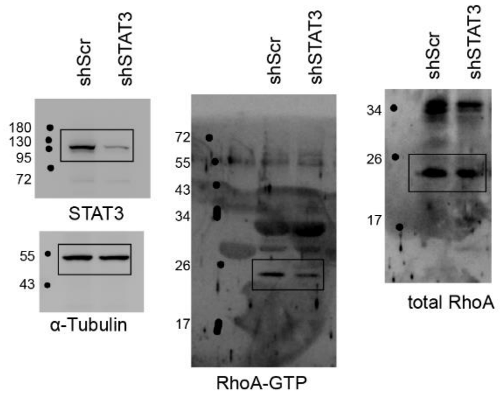

Fig. 4a

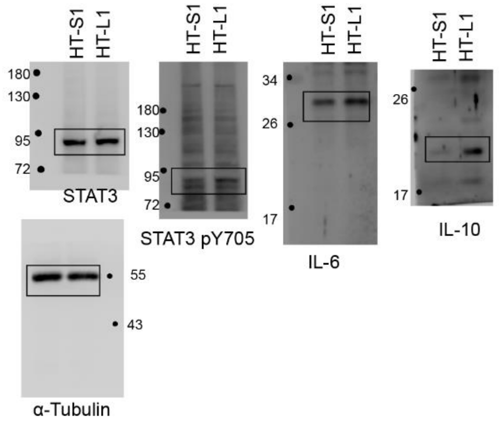

Fig. 4b

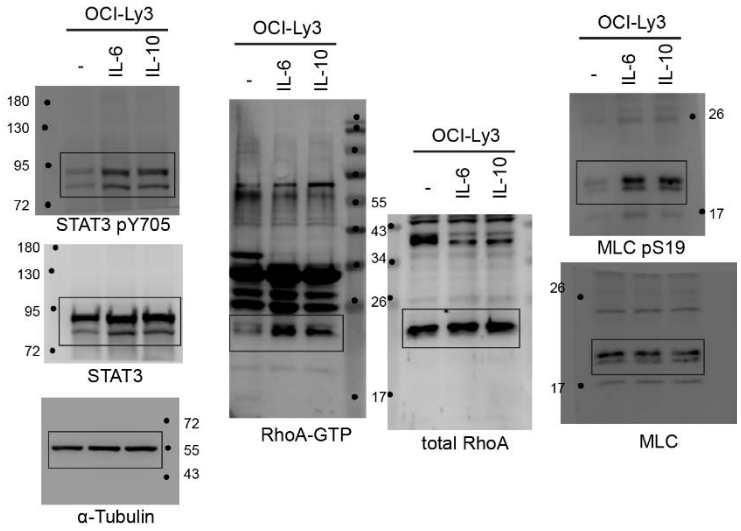

Supplementary Figure 8. Uncropped films of the experiments displayed in the figures and supplementary figures.

Fig. 5a

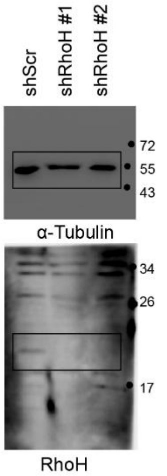

Fig. 5c

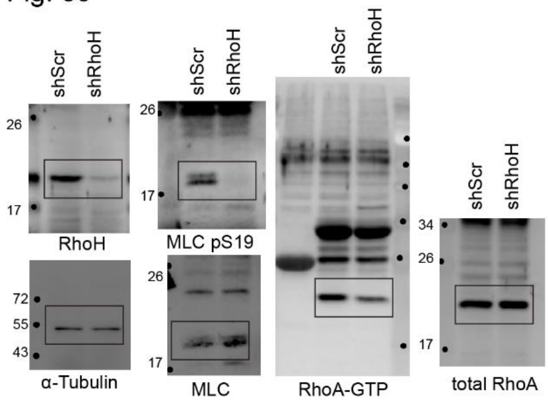

Fig. 5e

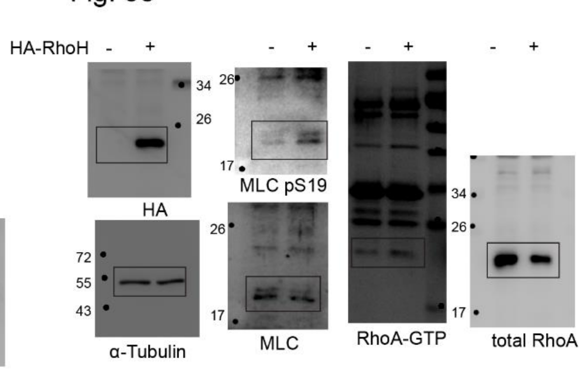

Fig. 5h

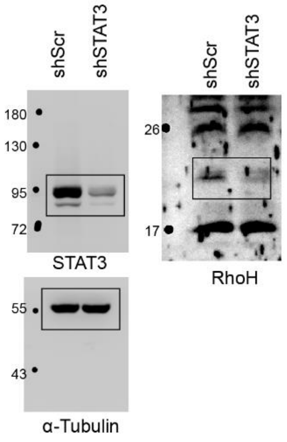

Fig. 5j

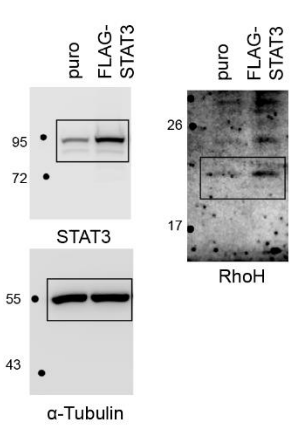

Fig. 5k

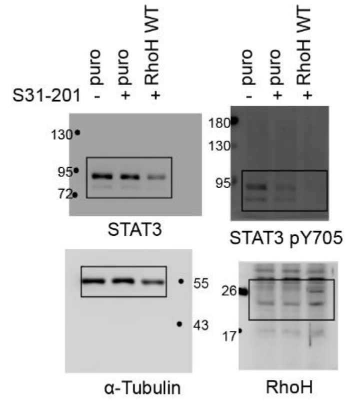

Fig. 5p

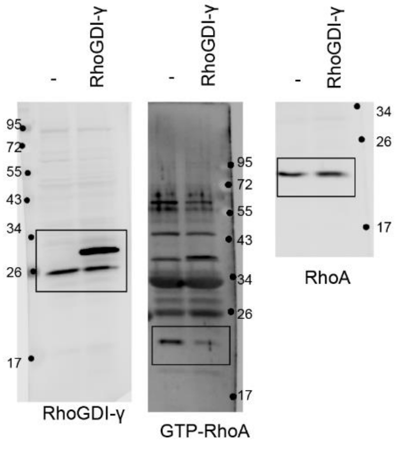

Fig. 5r

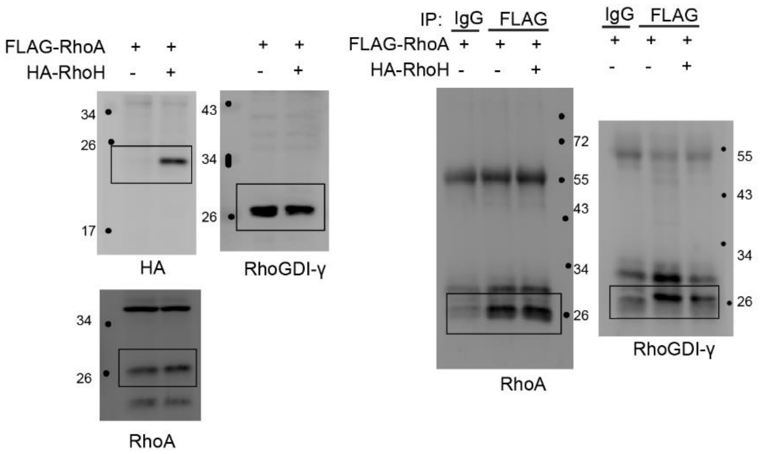

Supplementary Figure 8 (continued).

Fig. 5s

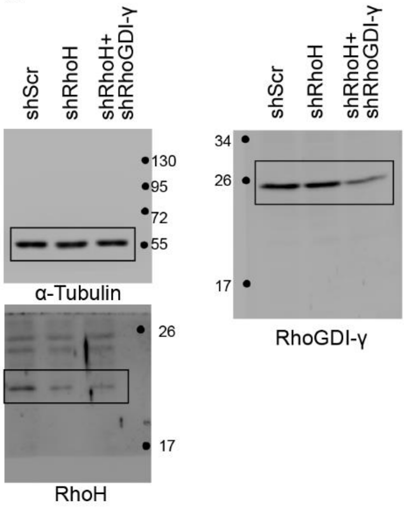

Fig. 6a

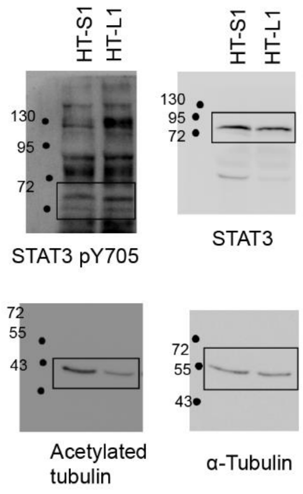

Fig. 6c

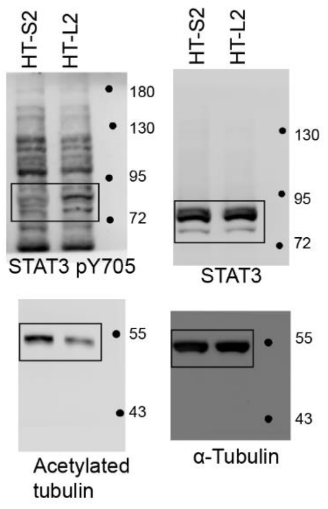

Fig. 6i

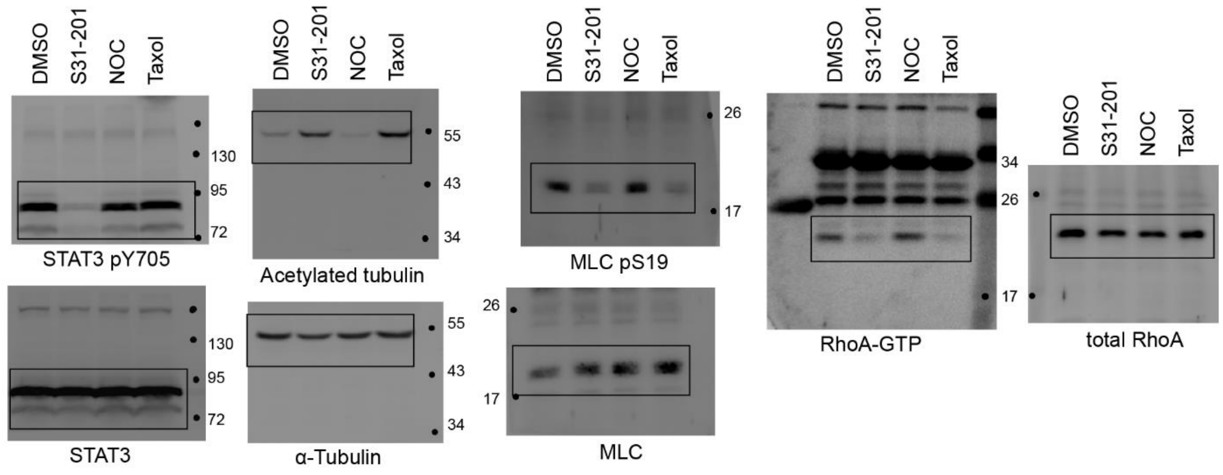

Fig. 6l

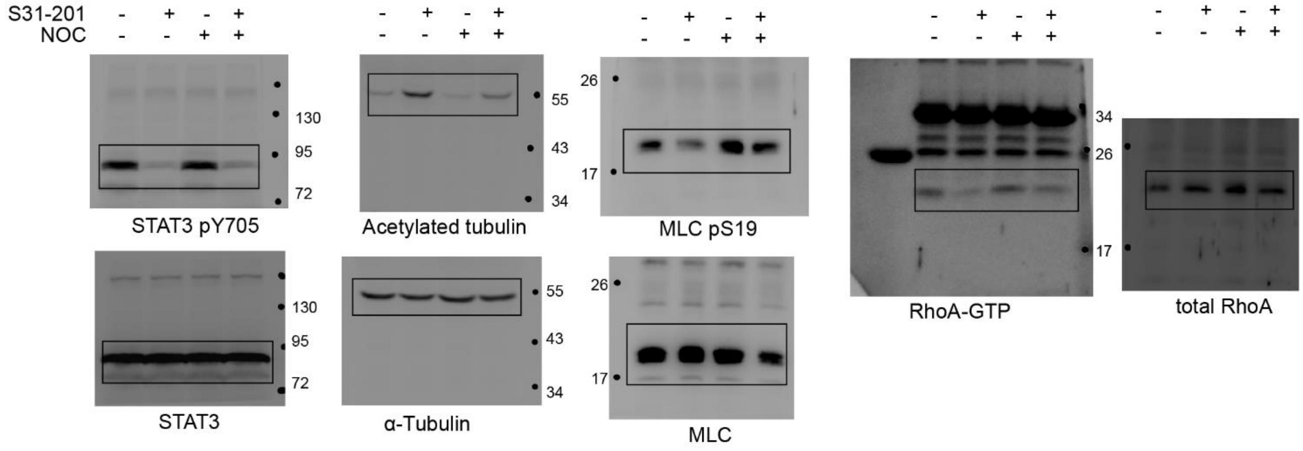

Supplementary Figure 8 (continued).

Supplementary Figures

Supplementary Fig. 2b

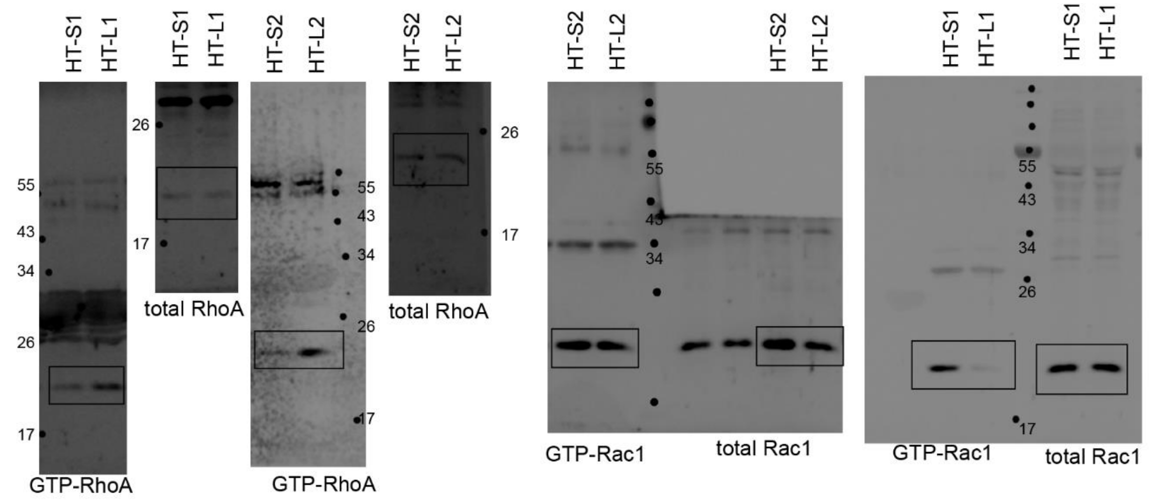

Supplementary Fig. 2b

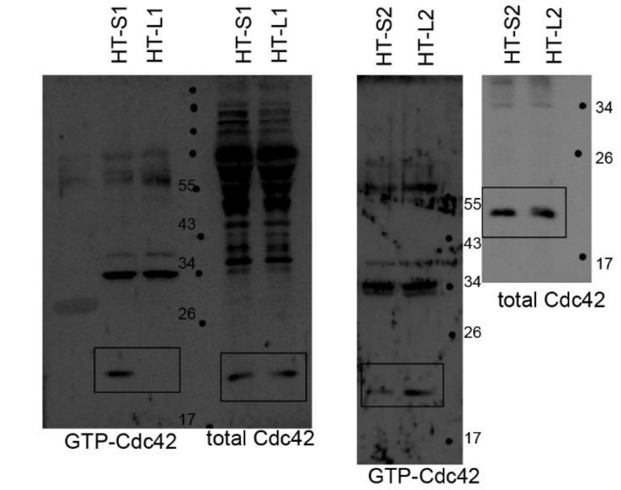

Supplementary Fig. 2e

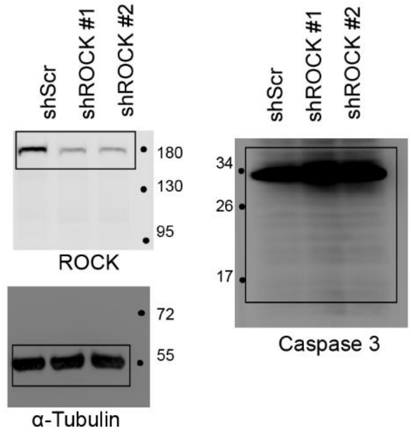

Supplementary Fig. 3a

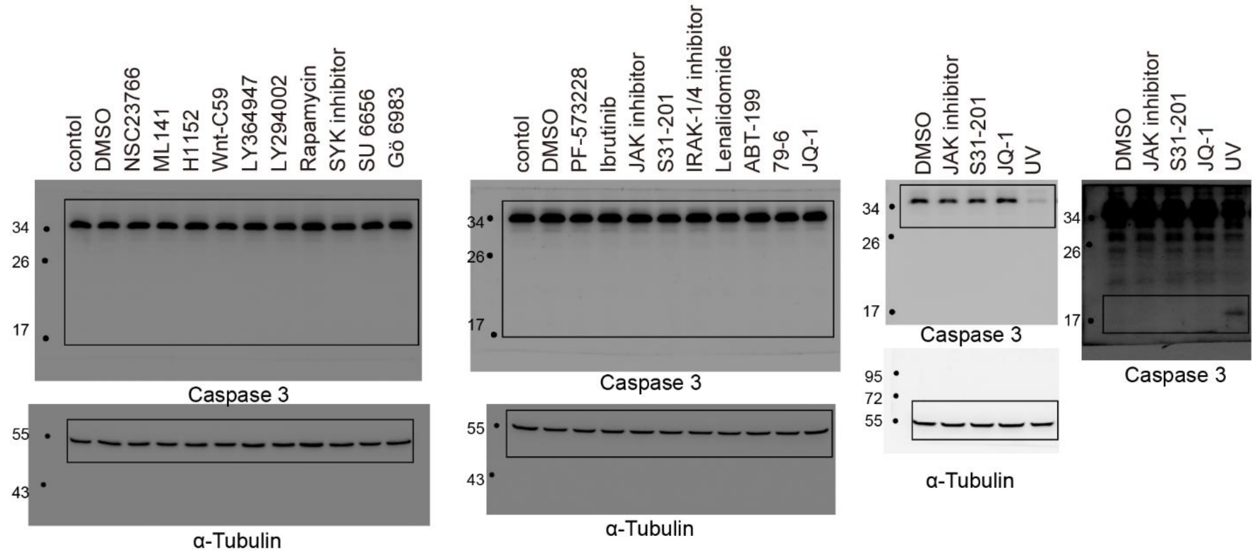

Supplementary Figure 8 (continued).

Supplementary Fig. 3c

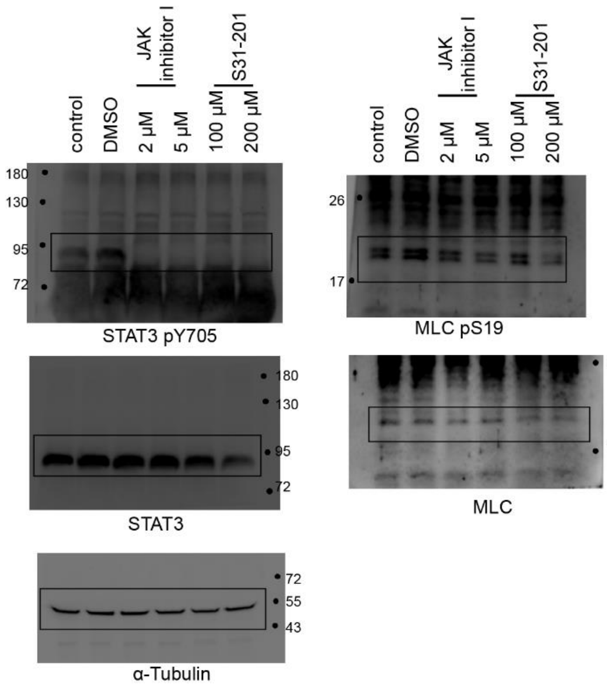

Supplementary Fig. 3e

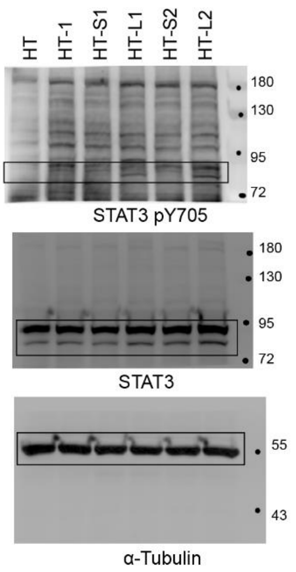

Supplementary Fig. 3f

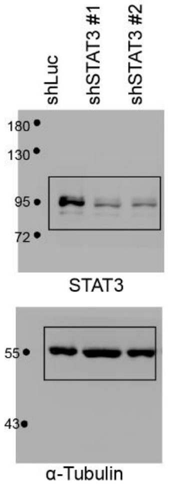

Supplementary Fig. 3i

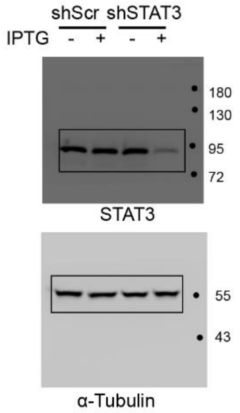

Supplementary Fig. 3k

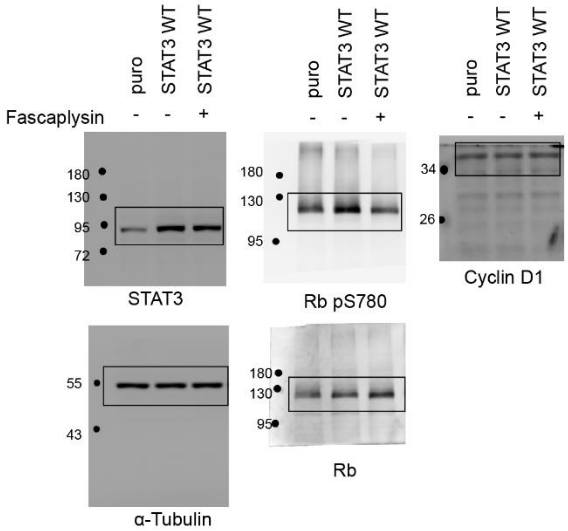

Supplementary Fig. 3n

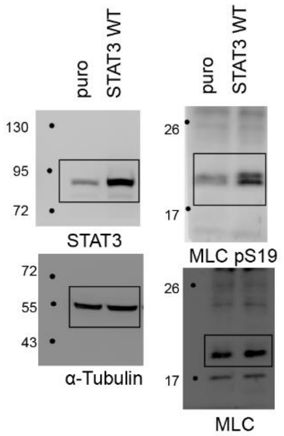

Supplementary Fig. 4a

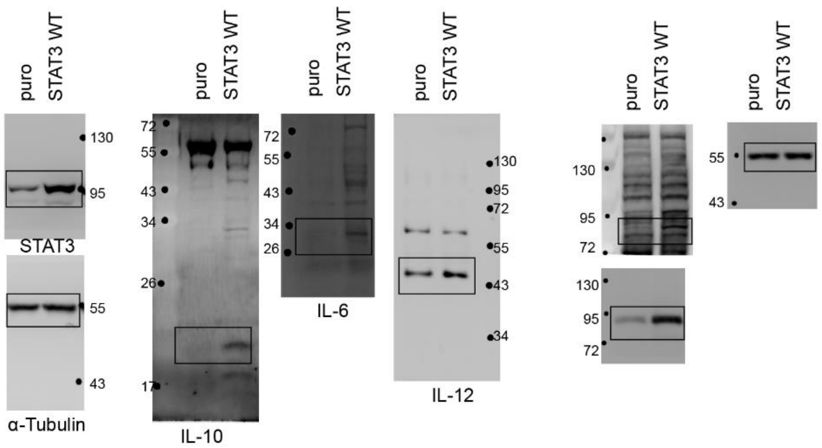

Supplementary Figure 8 (continued).

Supplementary Fig. 4c

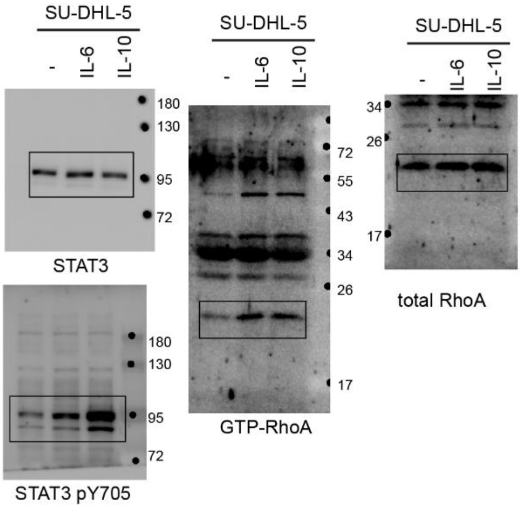

Supplementary Fig. 4g

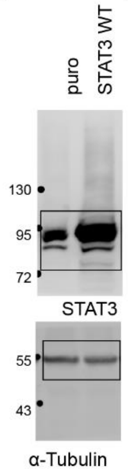

Supplementary Fig. 5h

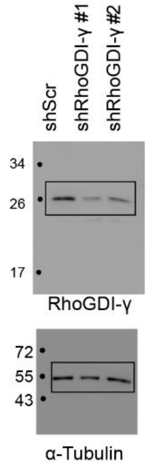

Supplementary Fig. 5i

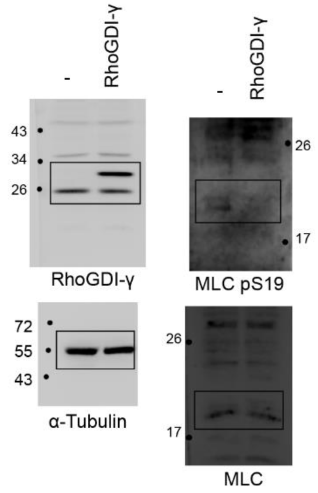

Supplementary Fig. 5j

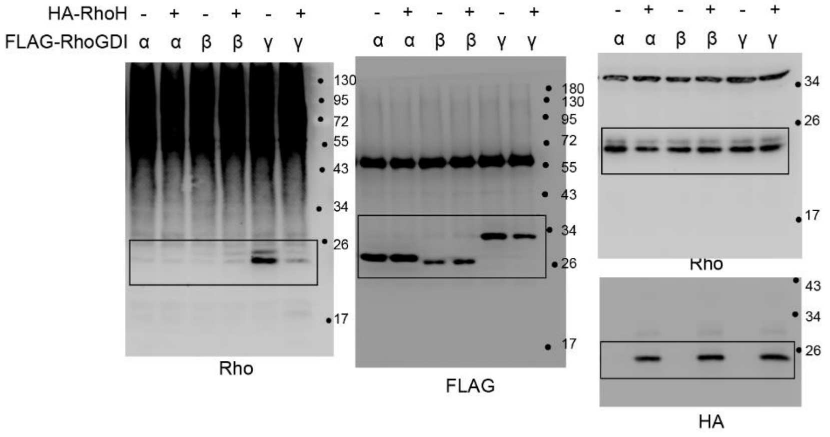

Supplementary Fig. 5k

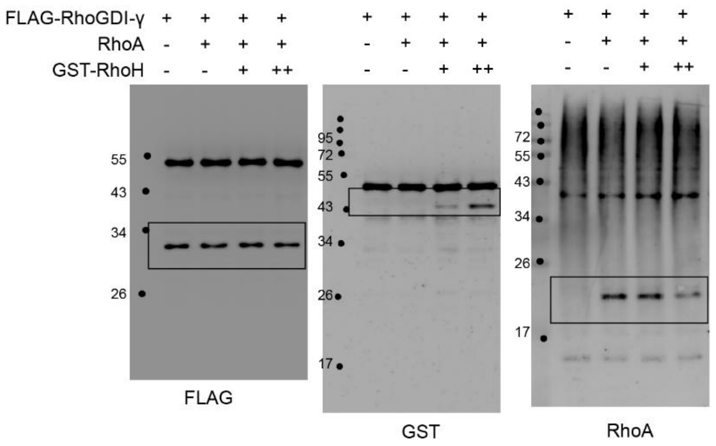

Supplementary Fig. 5m

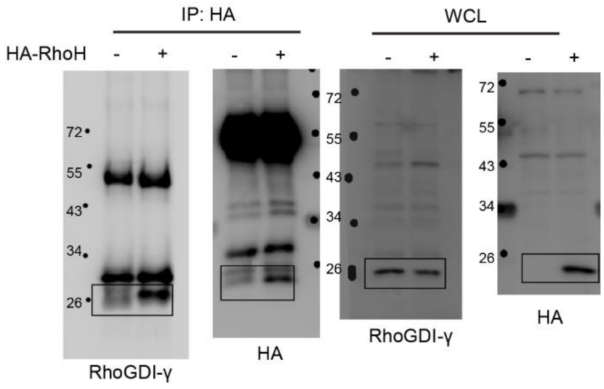

Supplementary Figure 8 (continued).

Supplementary Fig. 6b

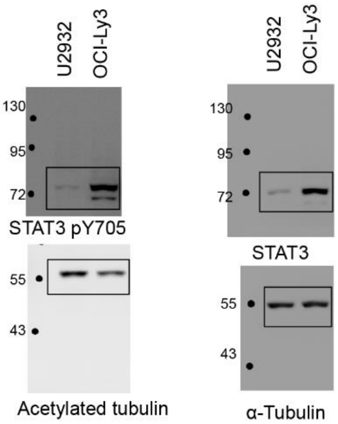

Supplementary Fig. 6c

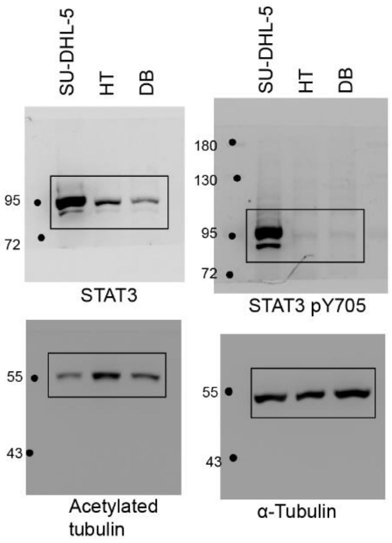

Supplementary Fig. 6d

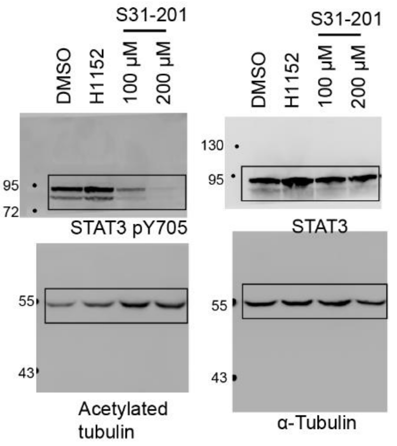

Supplementary Fig. 6g

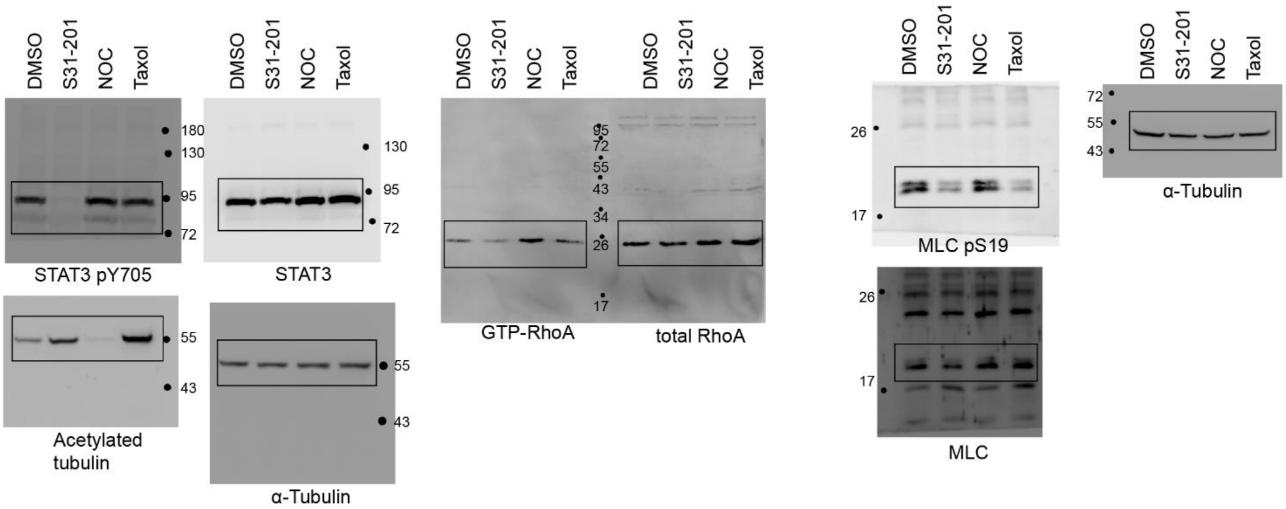

Supplementary Fig. 6j

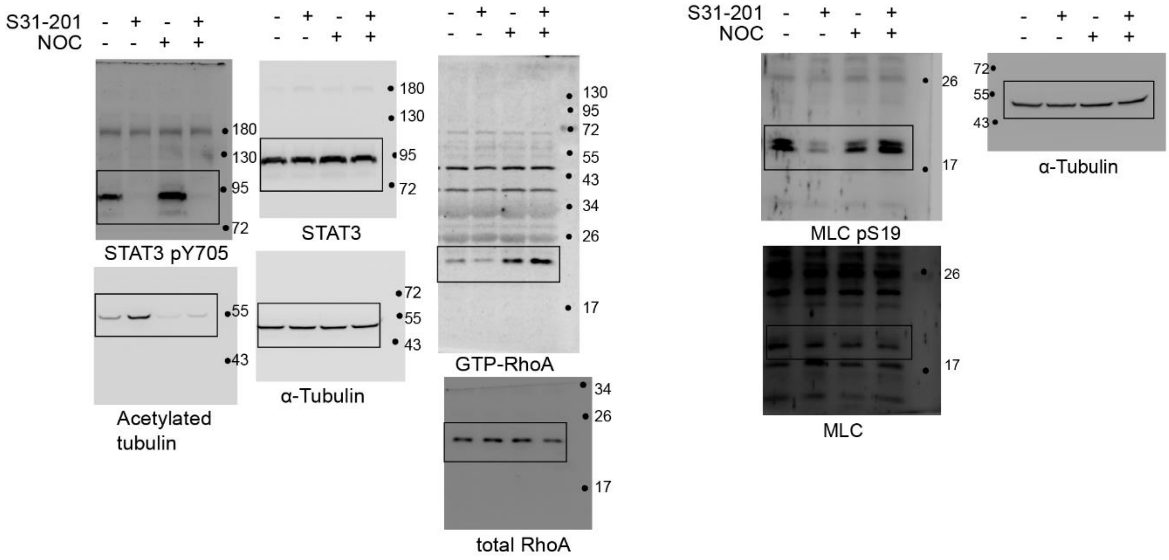

Supplementary Figure 8 (continued).

Supplementary Fig. 6m

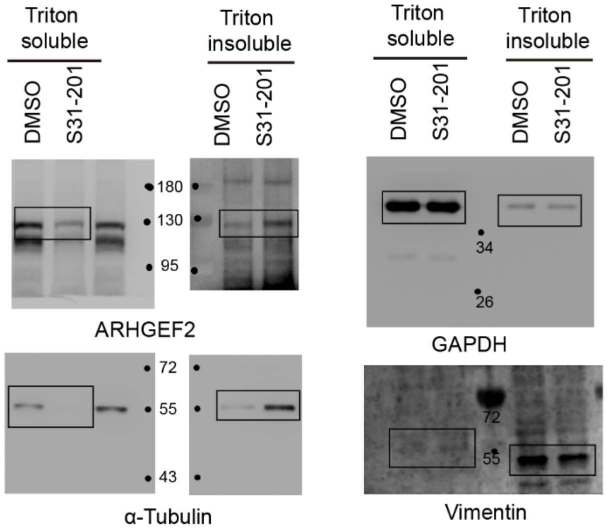

Supplementary Fig. 6o

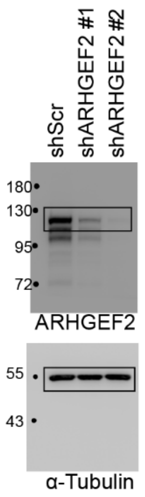

Supplementary Figure 8 (continued).

Supplementary Tables

Supplementary Table 1 Baseline characteristics of patients with diffuse large B-cell lymphoma

Variable	Value or Case number
Age in years; median (range)	67 (30-98)
Age ≧ 60 years; n (%)	48 (65.8%)
Males (%)	44 (60.3%)
Extranodal involvement; n (%)	46 (63.0%)
Ann Arbor Stage III/IV; n (%)	35 (47.9%)
IPI risk[a]; n (%)	
Low (score 0-1)	19 (27.5%)
Low-intermediate (score 2)	21 (30.4%)
High-intermediate (score 3)	12 (17.4%)
High (score 4-5)	17 (24.6%)
Activated B-Cell like histologic subtype; n (%)	40 (62.5%)
R-CHOP-like[b] chemotherapy; n (%)	61 (83.6%)

[a]IPI: International prognostic index bR-CHOP: Rituximab, cyclophosphamide, doxorubicin, vincristine, and prednisone

[b]R-CHOP: Rituximab, cyclophosphamide, doxorubicin, vincristine, and prednisone

Supplementary Table 2 The chi-squared test for DLBCL stages by MLC S19 phosphorylation

	Stage I	Stage II-IV	total	p-value
MLCpS19 Low[a]	7	5	12	0.035
MLCpS19 Hight[a]	2	10	12	
Total	9	15	24	

[a]: Cut off H score: 120

Supplementary Table 3 Information for the putative target pathways for DLBCL motility and corresponding inhibitors used in this study.

Signaling pathway	Candidate	Inhibitor	References
	Rac1	NSC23766	1, 2
	Cdc42	ML141	2, 3
	ROCK	H1152	2, 4
Wnt/PCP signaling pathway	Wnt signaling	Wnt-C59	5, 6
TGFβ signaling pathway	TGFβ	LY364947	7, 8
PI3K/Akt signaling pathway	PI3K	LY294002	9-11
B cell receptor signaling pathway			
PI3K/AKT/mTOR pathway	mTOR	rapamycin	12, 13
SYK-mediated signaling	SYK	SYK inhibitor	14-17
B cell receptor signaling pathway			
Integrin signaling pathway	Src	SU 6656	18-21
Growth factor-signaling pathways			
B cell receptor signaling pathway			
PKC-mediated signaling	PKC	Gö 6983	22, 23
Integrin signaling pathway	FAK	PF-573228	19, 24
B cell receptor signaling pathway	BTK	Ibrutinib	25, 26
JAK-STAT3 signaling pathway	JAK	JAK inhibitor I	27, 28
JAK-STAT3 signaling pathway	STAT3	S31-201	29, 30
MyD88 signaling pathway	IRAK-4	IRAK-1-4 Inhibitor I	31, 32
MyD88 signaling pathway	IRF-4	Lenalidomide	33, 34
Apoptosis signaling pathway	BCL-6	79-6	35, 36
Apoptosis signaling pathway	BCL-2	ABT-199	37, 38
	BET proteins	JQ1	39, 40

Supplementary Table 4 Information of inhibitors used in this study

Inhibitor	Target	Working concentration	Incorporation
79-6	BCL-6	50 μM	EMD Millipore (Billerica, MA)
Fascaplysin	cyclin D1	1 μM	EMD Millipore (Billerica, MA)
Blebbistatin	myosin II	10 μM	Sigma-Aldrich (St. Louis, MO)
ABT-199	BCL-2	1 μM	ApexBio Technology (Houston, TX)
Gö 6983	PKC	1 μM	ApexBio Technology (Houston, TX)
H1152	ROCK	2 μM	EMD Millipore (Billerica, MA)
Ibrutinib	BTK	2 μM	ApexBio Technology (Houston, TX)
IRAK-1-4 Inhibitor I	IRAK-4	5 μM	ApexBio Technology (Houston, TX)
JAK inhibitor I	pan-JAK	2 μM<br>5 μM in Supplementary Fig 3c,d	EMD Millipore (Billerica, MA)
JQ1	BET proteins	1 μM	ApexBio Technology (Houston, TX)
Lenalidomide	IRF-4	1 μM	ApexBio Technology (Houston, TX)
LY294002	PI3K	15 μM	Selleck Chemicals. (Houston, TX)
LY364947	TFGβ	10 μM	Sigma-Aldrich (St. Louis, MO)
ML141	cdc42	10 μM	BioVision (Milpitas, CA)
ML-7	MLCK	20 μM	Sigma-Aldrich (St. Louis, MO)
Nocodazole (Noc)	microtubule	3.3 μM	Sigma-Aldrich (St. Louis, MO)
NSC23766	Rac1	20 μM	Sigma-Aldrich (St. Louis, MO)
Paclitaxel (taxol)	microtubule	2 μM	Sigma-Aldrich (St. Louis, MO)
PF-573228	FAK	10 μM	ApexBio Technology (Houston, TX)
proteinase inhibitor (PI)	proteinase	1:800	Sigma-Aldrich (St. Louis, MO)
Rapamycin	mTOR	100 nM	EMD Millipore (Billerica, MA)
Ruxolitinib	JAK	1 μM	Novartis International AG (Basel, Switzerland).
S31-201	STAT3	100μM<br>200 μM in Supplementary Fig 3c, d and 6d	EMD Millipore (Billerica, MA)
SU 6656	Src	10 μM	EMD Millipore (Billerica, MA)
SYK inhibitor	SYK	10 μM	EMD Millipore (Billerica, MA)
Wnt-C59	Wnt signalling	20 μM	ApexBio Technology (Houston, TX)

Supplementary Table 5 Information of antibodies used in this study

Protein	Application (Dilution)	Antibody	Origin	Incorporation
Acetylated tubulin	WB (1:2000)	T6199	mouse mAb	Sigma-Aldrich (St. Louis, MO)
ARHGEF2 (GEF2)	WB (1:2000)	#4076	rabbit mAb	Cell Signaling Technology, Inc. (Danvers, MA)
Caspase 3	WB (1:1000)	#9662S	rabbit pAb	Cell Signaling Technology, Inc. (Danvers, MA)
CD19	CTC collection (1:50)	#555413	mouse mAb	BD Transduction Laboratories™ (Franklin Lakes, NJ)
CD20	IHC (1:200)	M0775	mouse mAb	Dako (Glostrup Denmark)
Cdc42	WB (1:1000)	A13981	mouse mAb	Thermo Fisher Scientific Inc. (Waltham, MA)
Cyclin D1	WB (1:1000)	#2978	rabbit mAb	Cell Signaling Technology, Inc. (Danvers, MA)
FLAG	WB (1:1000), IP	F1804	mouse mAb	Sigma-Aldrich (St. Louis, MO)
HA	WB (1:1000), IP	901503	mouse mAb	BioLegend, Inc. (San Diego, CA)
IL-10	WB (1:1000), neutralising	ab34843	rabbit pAb	Abcam Plc. (Cambridge, UK)
IL-12	WB (1:1000), neutralising	MAB1570	mouse mAb	R&D Systems, Inc. (Minneapolis, MN)
IL-6	WB (1:1000), neutralising	ab11449	mouse mAb	Abcam Plc. (Cambridge, UK)
MLC	WB (1:2000)	#3672	rabbit pAb	Cell Signaling Technology, Inc. (Danvers, MA)
MLC pS19	WB (1:1000), IHC (1:50)	#3675	mouse mAb	Cell Signaling Technology, Inc. (Danvers, MA)
Rac1	WB (1:1000)	PA1-091X	mouse mAb	Thermo Fisher Scientific Inc. (Waltham, MA)
Rb	WB (1:1000)	#9309	mouse mAb	Cell Signaling Technology, Inc. (Danvers, MA)
Rb pS780	WB (1:1000)	#8180S	rabbit mAb	Cell Signaling Technology, Inc. (Danvers, MA)
RhoA	WB (1:1000)	sc-418	mouse mAb	Santa Cruz Biotechnology Inc. (Dallas, TX)
RhoGDI-γ	WB (1:1000)	ab157493	rabbit pAb	Abcam Plc. (Cambridge, UK)
RhoH	WB (1:1000), IHC (1:200)	ab118507	rabbit pAb	Abcam Plc. (Cambridge, UK)
ROCK1	WB (1:1000)	#4035	rabbit mAb	Cell Signaling Technology, Inc. (Danvers, MA)
STAT3	WB (1:5000), ChIP	#9139	mouse mAb	Cell Signaling Technology, Inc. (Danvers, MA)
STAT3 pY705	WB (1:1000), IHC (1:200)	#9145	rabbit mAb	Cell Signaling Technology, Inc. (Danvers, MA)
α-tubulin	WB (1:5000)	T6793	mouse mAb	Sigma-Aldrich (St. Louis, MO)
Abbreviations: ChIP, chromatin immunoprecipitation;; IF, immunofluorescence; IHC, immunohistochemistry; IP, immunoprecipitation; mAb: monoclonal antibody; pAb, polyclonal antibody; WB, western blot				

Supplementary Table 6 Information of shRNA sequences

Gene name	shRNA target seguence
Scr	TTTATGCAATAAGACCTATTC
ARHGDIG #1	CGTGGACAAGACCGTCTACAT
ARHGDIG #2	CCCAGGAGTATGAGTTTGTGA
ARHGEF2 #1	CGATGCCCTGTACTTGAGTTT
ARHGEF2 #2	CCCAACCTGCAATGTGACTAT
RhoH #1	CCATAACTCATTCCTGAACTT
RhoH #2	GAGTGCTCAGCCCTTAGCAAT
ROCK1 #1	GCATTCCAAGATGATCGTTAT
ROCK1 #2	GAGGTAAATGAACACAAAGTA
STAT3 #1	GCAAAGAATCACATGCCACTT
STAT3 #2	GCACAATCTACGAAGAATCAA
Oligonucleotides for IPTG- Controlled shRNA	
shScr	CCGGTTTATGCAATAAGACCTATTCCTCGAGGAATAGGTCTTATTGCATAAATTTTT
shSTAT3	CCGGGCACAATCTACGAAGAATCAACTCGAGTTGATTCTTCGTAGATTGTGCTTTTT

Supplementary Table 7 Information of PCR primers used in this study

Primers for q-PCR			Primers for q-PCR		
gene name		sequence (5'-3')	RHOD	F	GAAGACGTCGCTGCTGATG
ARHGAP1	F	CGGATGAAATGCCTGACTTC		R	ATGTGGAGGTGCACAGGTTT
	R	CTTCCGCCCATACTTGTCAT	RHOG	F	TTAAGGAGGCCTGCAGAGAA
ARHGDIA	F	CATTTAGTGCGGGAGGGAT		R	GTCAGTAGCGGAAAATGGGA
	R	CTCGTTCTCCGCTGCAAT	RHOH	F	ACATTTCTTCGGCATTCTGC
ARHGEF11	F	GATTCTGGAACCGTGGAGAC		R	ACTCCAAGAAGTGGCAAAGG
	R	TCTCCCAGAGAAGACAGGCT	RHOQ	F	CTTCGACCACTACGCAGTCA
EZR	F	TGATGGACCAGCACAAACTT		R	AGGAAGACATCGGTCATTGG
	R	CAGGTCCTGAGCAATCTTCA	RHOT1	F	ATGAAGAAAGACGTGCGGAT
GNAI2	F	GGAGGTGAAGTTGCTGCTGT		R	TGGAATGGTGATTTCTTCTGC
	R	ATGGACTGGATGGTGTTGCT	RHOT2	F	GGACTACTCAGAAGCCGAGC
LPAR3	F	GGAGGACACCCATGAAGCTA		R	CAGTGGGATCCACTTAGTTCG
	R	GGAACCACCTTTTCACATGC	RHOV	F	CAGCCTCATCGTCAGCTACA
MYBPH	F	AGTGAGTTCTGCAGGGGCT		R	GCTGTGTCCCAGAGCTCAAT
	R	ATTTGCAGGTTGACCGTCTC	RND1	F	ACCTGCGAACAGACCTGAGT
MYL2	F	CTTCCACCATGGCACCTAAG		R	GGTAGATTTCTGCACCCAGC
	R	GTCAATGAAGCCATCCCTGT	RND2	F	GCGAGCTTTGAGATCGACA
PIP4K2A	F	TTCGTAGCGCAGAAAGTGAA		R	GCACAGCATCAGAATCAGGA
	R	TGGCATCAACATAACAGGGA	RND3	F	GGCCAGTTTTGAAATCGACA
PKN1	F	TTACAGCCGAAGCGGAAG		R	TCAAAGCAAATCAGCACAGC
	R	CTGCCCCACCACTGTGTTAT	ROCK1	F	GGGGACAGTTTTGAGACTCG
PPP1R12A	F	CCAATGTGGACGGACTCAC		R	GGCAGGAAAATCCAAATCAT
	R	GGAAGCTGCTGCATGTAGTG	RTKN	F	GATGCAGGACAGATTGCACA
PPP1R12B	F	TCTGGCAAGAGGTGCTGATA		R	TCCGGATCTCATGGTCTAGC
	R	CCTCGTTGTCTTGCTGGTTT	STAT3	F	CCTCTGCCGGAGAAACAG
RHOA	F	GTCTGGTCTTCAGCTACCCG		R	CTGCTCCAGGTACCGTGTGT
	R	AGGCTCCATCACCAACAATC	WASF1	F	GCTATGGCTGCAAAATCGTT
RHOB	F	CCAAGCCTACGACTACCTCG		R	ATGCCTCTAGGCAGTGCTGT
	R	TCATAGCACCTTGCAGCAGT	Primers for ChIP		
RHOBTB1	F	TCCAGCTGTGAGCAGAGTGT	gene name		sequence (5'-3')
	R	GTCACCCACGACCACACAT	CON	F	AAGTTTCTCTTTAATCTCAGCA
RHOBTB2	F	CGTGCTCAGCAGGAAGAGAT		R	AAGACAAAAATGACACTAAAACT
	R	CGTTTGGCCTTTCATAATCC	RHOH	F	TGGAAGTCTTGTGAACTTGGT
RHOC	F	CGGAAGCCTTGACTTCATCT		R	GAGGAAAGAACATTCCACGTA
	R	CCAACGATCACCAGCTTCTT			

Supplementary references

1. Bid, H. K., Roberts, R. D., Manchanda, P. K. & Houghton, P. J. RAC1: an emerging therapeutic option for targeting cancer angiogenesis and metastasis. *Mol. Cancer Ther.* **12**, 1925-1934 (2013).
2. Sahai, E. & Marshall, C. J. RHO-GTPases and cancer. *Nat. Rev Cancer* **2**, 133-142 (2002).
3. Calvo, F. et al. RasGRF suppresses Cdc42-mediated tumour cell movement, cytoskeletal dynamics and transformation. *Nat. Cell Biol.* **13**, 819-826 (2011).
4. Matsuoka, T. & Yashiro, M. Rho/ROCK signaling in motility and metastasis of gastric cancer. *World J. Gastroenterol.* **20**, 13756-13766 (2014).
5. Schlessinger, K., Hall, A. & Tolwinski, N. Wnt signaling pathways meet Rho GTPases. *Genes Dev.* **23**, 265-277 (2009).
6. Wang, Y. Wnt/Planar cell polarity signaling: a new paradigm for cancer therapy. *Mol. Cancer Ther.* **8**, 2103-2109 (2009).
7. Heldin, C. H., Vanlandewijck, M. & Moustakas, A. Regulation of EMT by TGFbeta in cancer. *FEBS Lett.* **586**, 1959-1970 (2012).
8. Papageorgis, P. TGFbeta Signaling in Tumor Initiation, Epithelial-to-Mesenchymal Transition, and Metastasis. *J. Oncol.* **2015**, 587193 (2015).
9. Arcaro, A. & Guerreiro, A. S. The phosphoinositide 3-kinase pathway in human cancer: genetic alterations and therapeutic implications. *Curr. Genomics* **8**, 271-306 (2007).
10. Pfeifer, M. & Lenz, G. PI3K/AKT addiction in subsets of diffuse large B-cell lymphoma. *Cell Cycle* **12**, 3347-3348 (2013).
11. Roschewski, M., Staudt, L. M. & Wilson, W. H. Diffuse large B-cell lymphoma-treatment approaches in the molecular era. *Nat. Rev. Clin. Oncol.* **11**, 12-23 (2014).
12. Gulhati, P. et al. mTORC1 and mTORC2 regulate EMT, motility, and metastasis of colorectal cancer via RhoA and Rac1 signaling pathways. *Cancer Res.* **71**, 3246-3256 (2011).
13. Zhou, H. & Huang, S. Role of mTOR signaling in tumor cell motility, invasion and metastasis. *Curr. Protein Pept. Sci.* **12**, 30-42 (2011).
14. Chen, L. et al. SYK-dependent tonic B-cell receptor signaling is a rational treatment target in diffuse large B-cell lymphoma. *Blood* **111**, 2230-2237 (2008).
15. Cheng, S. et al. SYK inhibition and response prediction in diffuse large B-cell lymphoma. *Blood* **118**, 6342-6352 (2011).
16. Krisenko, M. O. & Geahlen, R. L. Calling in SYK: SYK's dual role as a tumor promoter and tumor suppressor in cancer. *Biochim. Biophys. Acta* **1853**, 254-263 (2015).
17. Luangdilok, S. et al. Syk tyrosine kinase is linked to cell motility and progression in squamous cell carcinomas of the head and neck. *Cancer Res.* **67**, 7907-7916 (2007).
18. Hollmann, C. A. et al. Therapeutic implications of Src independent calcium mobilization in diffuse large B-cell lymphoma. *Leuk. Res.* **34**, 585-593 (2010).
19. Hood, J. D. & Cheresh, D. A. Role of integrins in cell invasion and migration. *Nat. Rev. Cancer* **2**, 91-100 (2002).
20. Ke, J. et al. Anomalous constitutive Src kinase activity promotes B lymphoma survival and growth. *Mol. Cancer* **8**, 132 (2009).
21. Yeatman, T. J. A renaissance for SRC. *Nat. Rev. Cancer* **4**, 470-480 (2004).

22. Garg, R. et al. Protein kinase C and cancer: what we know and what we do not. *Oncogene* **33**, 5225-5237 (2014).
23. Griner, E. M. & Kazanietz, M. G. Protein kinase C and other diacylglycerol effectors in cancer. *Nat. Rev. Cancer* **7**, 281-294 (2007).
24. Sulzmaier, F. J., Jean, C. & Schlaepfer, D. D. FAK in cancer: mechanistic findings and clinical applications. *Nat. Rev. Cancer* **14**, 598-610 (2014).
25. Hendriks, R. W., Yuvaraj, S. & Kil, L. P. Targeting Bruton's tyrosine kinase in B cell malignancies. *Nat. Rev. Cancer* **14**, 219-232 (2014).
26. Rickert, R. C. New insights into pre-BCR and BCR signalling with relevance to B cell malignancies. *Nat. Rev. Immunol* **13**, 578-591 (2013).
27. Beck, D. et al. Synthetic Lethal Screen Demonstrates That a JAK2 Inhibitor Suppresses a BCL6-dependent IL10RA/JAK2/STAT3 Pathway in High Grade B-cell Lymphoma. *J. Biol. Chem.* **291**, 16686-16698 (2016).
28. Gupta, M. et al. Elevated serum IL-10 levels in diffuse large B-cell lymphoma: a mechanism of aberrant JAK2 activation. *Blood* **119**, 2844-2853 (2012).
29. Ok, C. Y. et al. Clinical implications of phosphorylated STAT3 expression in De Novo diffuse large B-cell lymphoma. *Clin. Cancer Res.* **20**, 5113-5123 (2014).
30. Scuto, A. et al. STAT3 inhibition is a therapeutic strategy for ABC-like diffuse large B-cell lymphoma. *Cancer Res.* **71**, 3182-3188 (2011).
31. Kuppers, R. IRAK4 inhibition to shut down TLR signaling in autoimmunity and MyD88-dependent lymphomas. *J. Exp. Med.* **212**, 2184 (2015).
32. Ngo, V. N. et al. Oncogenically active MYD88 mutations in human lymphoma. *Nature* **470**, 115-119 (2011).
33. Care, M. A. et al. SPIB and BATF provide alternate determinants of IRF4 occupancy in diffuse large B-cell lymphoma linked to disease heterogeneity. *Nucleic Acids Res.* **42**, 7591-7610 (2014).
34. Salaverria, I. et al. Translocations activating IRF4 identify a subtype of germinal center-derived B-cell lymphoma affecting predominantly children and young adults. *Blood* **118**, 139-147 (2011).
35. Cerchietti, L. C. et al. BCL6 repression of EP300 in human diffuse large B cell lymphoma cells provides a basis for rational combinatorial therapy. *J. Clin. Invest.* **120**, 4569-4582 (2010).
36. Ueda, C., Akasaka, T. & Ohno, H. Non-immunoglobulin/BCL6 gene fusion in diffuse large B-cell lymphoma: prognostic implications. *Leuk. Lymphoma* **43**, 1375-1381 (2002).
37. Friedberg, J. W. Double hit diffuse large B-cell lymphomas: diagnostic and therapeutic challenges. *Chin. Clin. Oncol.* **4**, 9 (2015).
38. Monni, O., Franssila, K., Joensuu, H. & Knuutila, S. BCL2 overexpression in diffuse large B-cell lymphoma. *Leuk. Lymphoma* **34**, 45-52 (1999).
39. Chapuy, B. et al. Discovery and characterization of super-enhancer-associated dependencies in diffuse large B cell lymphoma. *Cancer Cell* **24**, 777-790 (2013).
40. Mottok, A. & Gascoyne, R. D. Bromodomain inhibition in diffuse large B-cell lymphoma-giving MYC a brake. *Clin. Cancer Res.* **21**, 4-6 (2015).
