# Supplementary material for: STAT3-coordinated migration facilitates the dissemination of diffuse large B-cell lymphomas
Source: Nat Commun. 2018 Sep 12;9:3696. doi: 10.1038/s41467-018-06134-z (PMC6135800; doi:10.1038/s41467-018-06134-z)
Supplement: Supplementary file 2 — Description of Additional Supplementary Files [file 41467_2018_6134_MOESM2_ESM.pdf]

Description of Additional Supplementary Files

File Name: Supplementary Data 1
Description: Upregulated and downregulated genes in cDNA microarray analysis of HT-Lx vs HT-Sx, HT-L1 vs HT-S1, and HT-L2 vs HT-S2.

File Name: Supplementary Data 2
Description: Information of gene signatures for GSEA used in this study.

File Name: Supplementary Movie 1
Description: **Amoeboid movement in DLBCL cell lines and OEC-M1 cells.** Time-lapse video microscopy of SU-DHL-5, HT, DB, OCI-Ly3 and U2932 cells in 3D collagen gels and OEC-M1 in 2.5 D collagen gels. The duration of image capture was 6 hours for DLBCL cell lines or 16 hours for OECM1 cells.

File Name: Supplementary Movie 2
Description: **Amoeboid movement in HT-Sx, HT-Lx, HT-S1, HT-L1, HT-S2 and HT-L2 sublines.** Time-lapse video microscopy of HT-Sx, HT-Lx, HT-S1, HT-L1, HT-S2 and HT-L2 sublines in 3D collagen gels. The duration of image capture was 6 hours.

File Name: Supplementary Movie 3
Description: **Amoeboid movement in SU-DHL-5 or OCI-Ly3 cells treated by inhibitors.** Time-lapse video microscopy of SU-DHL-5 or OCI-Ly3 cells treated by inhibitors (1 μM H1152, 2 μM pan-JAK inhibitor I, 100 μM S31-201 or 1 μM JQ1) or control DMSO in 3D collagen gels. The duration of image capture was 6 hours.

File Name: Supplementary Movie 4
Description: **Amoeboid movement in RhoH knockdown SU-DHL-5 cells.** Time-lapse video microscopy of SU-DHL-5 cells expressing shRNAs specific to RhoH (shRhoH; clones #1, #2) or scramble (shScr) in 3D collagen gels. The duration of image capture was 6 hours.

File Name: Supplementary Movie 5
Description: **Amoeboid movement in SU-DHL-5 cells treated by NOC or Taxol.** Time-lapse video microscopy of SU-DHL-5 cell treated by inhibitors (3.3 μM NOC or 2 μM Taxol) or control DMSO in 3D collagen gels. The duration of image capture was 6 hours.
